# Endophytic fungal communities of *Polygonum acuminatum* and *Aeschynomene fluminensis* are influenced by soil mercury contamination

**DOI:** 10.1371/journal.pone.0182017

**Published:** 2017-07-25

**Authors:** William Pietro-Souza, Ivani Souza Mello, Suzana Junges Vendruscullo, Gilvan Ferreira da Silva, Cátia Nunes da Cunha, James Francis White, Marcos Antônio Soares

**Affiliations:** 1 Department of Botany and Ecology, Laboratory of Biotechnology and Microbial Ecology, Institute of Biosciences, Federal University of Mato Grosso, Cuiabá, Mato Grosso, Brazil; 2 Department of Botany and Ecology, Institute of Biosciences, Federal University of Mato Grosso, Brazil; 3 Embrapa Amazônia Ocidental, Manaus, Amazonas, Brazil; 4 Department of Plant Biology, Rutgers University, New Brunswick, NJ, United States of America; University of Vigo, SPAIN

## Abstract

The endophytic fungal communities of *Polygonum acuminatum* and *Aeschynomene fluminensis* were examined with respect to soil mercury (Hg) contamination. Plants were collected in places with and without Hg^+2^ for isolation and identification of their endophytic root fungi. We evaluated frequency of colonization, number of isolates and richness, indices of diversity and similarity, functional traits (hydrolytic enzymes, siderophores, indoleacetic acid, antibiosis and metal tolerance) and growth promotion of *Aeschynomene fluminensis* inoculated with endophytic fungi on soil with mercury. The frequency of colonization, structure and community function, as well as the abundant distribution of taxa of endophytic fungi were influenced by mercury contamination, with higher endophytic fungi in hosts in soil with mercury. The presence or absence of mercury in the soil changes the profile of the functional characteristics of the endophytic fungal community. On the other hand, tolerance of lineages to multiple metals is not associated with contamination. *A*. *fluminensis* depends on its endophytic fungi, since plants free of endophytic fungi grew less than expected due to mercury toxicity. In contrast plants containing certain endophytic fungi showed good growth in soil containing mercury, even exceeding growth of plants cultivated in soil without mercury. The data obtained confirm the hypothesis that soil contamination by mercury alters community structure of root endophytic fungi in terms of composition, abundance and species richness. The inoculation of *A*. *fluminensis* with certain strains of stress tolerant endophytic fungi contribute to colonization and establishment of the host and may be used in processes that aim to improve phytoremediation of soils with toxic concentrations of mercury.

## Introduction

Mercury is a metal of natural occurrence where concentrations in the environment have increased over the years since the beginning of the industrial period [1]. The use in the artisanal beneficiation of gold considerably contributed to this increase in environmental mercury. In Brazil, mercury contamination occurs mainly in areas located in the Amazon region [2], but an increase in mercury concentrations in other biomes, as in the Pantanal, the worlds largest humid freshwater area, has been reported in Brazil [3,4] mercury contamination in aligators [5], fish [6] and molluscs [7] is caused by the poor mining practices in gold mines in the Pantanal.

The influence of mercury on the Pantanal microbiota is not yet known. However, it is known that prolonged exposure to toxic agents, such as heavy metals, alters the structure and function of microbial communities, selecting species capable of tolerating and, in certain cases, metabolizing toxic agents, because of adaptive mechanisms of tolerance [8,9]. The high mercury concentrations, cadmium and zinc modified the composition and abundance of the soil fungi community in the United States [10], Finland [11] and Belgium [12]. Meanwhile, the influence of heavy metals, including mercury, on the endophytic fungi community is rare, especially about the composition and function of resistant species able to help the hosts colonize contaminated soils.

Endophytic fungi colonize plant tissues internally without causing apparent symptoms of colonization and disease [13,14]. Plants that colonize places contaminated with heavy metals, especially Cd^2+^ Pb^2+^ and Zn^2+^, may harbor specific communities of contaminant tolerant endophytic fungi and may reduce the adverse effect of contamination in several hosts, such as *Zea mays* [15] *Portuca oleraceae* [16], *Verbascum lychnitis* [17], *Clethra barbinervis* [18], *Brassica napus* [19] and *Solanum nigrum* [20], consistent with the habitat-adapted symbiosis hypothesis [21].

Endophytes often promote the growth of their host by several mechanisms, including phytohormone synthesis [22], macro and micro nutrient solubilization [23,24], enzyme production [25,26], host protection against phytopathogens and herbivores [27,28] and in the mitigation of effects caused by exposure to extreme abiotic factors, such as heavy metals [19], salinity [29] and drought. This set of mechanisms represents different functional traits involved in promoting host plant growth. The impact of the endophytic community is not limited only to hosts. The endophytic community is also important in its influence on the structure and function of the soil microbial community [30,31].

The microbial features that relate to phosphorus solubilization, iron sequestration and phytohormone production are important in plant growth processes and host resistance to heavy metals [32]. In order to solubilize phosphate, the microorganisms produce and secrete organic acids that modify the pH and influence on solubility of heavy metals. This mechanism of phosphate solubilization increases plant biomass and mobilizes heavy metals [33,34].

Siderophores molecules are involved in the iron chelation process and can interact with heavy metals and influence mobility and absorption of heavy metals by the plant [35,36]. The synthesis of phytohormone, such as indoleacetic acid (IAA), stimulates the radicular growth providing the plant a greater acquisition to nutrient and heavy metals [37].

Endophytes have been associated with metal hyperaccumulating plants such as *Phragmites autralis* [38]. In spite of this association, the mechanisms by which endophytes increase metal removal are still unknown. Our hypothesis is based on the premise that mercury influences the structure and function of the metal resistant endophytic fungi community and that this microbial community mitigates mercury toxicity by promoting host plant growth. Thus, in this study we try to answer the following questions: 1) Does soil contamination with mercury cause changes in the community of endophytic fungi resistant to mercury? 2) Is the pattern of mercury resistance of strains determined by the environment? 3) Do mercury resistant endophytic fungi present resistance to other heavy metals? Do mercury-resistant endophytic fungi promote host plant growth on soil containing metal at toxic levels?

## Material and methods

### Characterization of the sampling site and selection of plant species

Plants were sampled in Poconé—Mato Grosso, Brazil, in areas characterized as wetland, with the influence of the low amplitude flood pulse [39,40]. This is the main mining area for gold (*garimpos*) in the Pantanal north, with a history of exploration since the 18th century and with the highest gold production during the 1980s [41].

Our studies did not involve endangered or protected species. The collection authorization (number 24237–3) was granted by the Chico Mendes Institute for Biodiversity Conservation (ICMBio) of the Ministry of the Environment (MMA).

The soil samples were collected in 10 subsamples at depth 0–10 cm for physicochemical analysis to define the areas with contamination (+Hg site—S"16°15'42.7" W"056°38'43.6") and without contamination (-Hg site 1—S"16°21'19.7"W"056°20'13.9" and–Hg site 2 S"16°15'51.3"W"056°38'54.3"). The area contaminated with mercury was used in the past to extract the gold collected in the mines located in the city of Poconé.

The frequency of species was determined using the point plot method [42]. The species *Polygonum acuminatum* Kunth. and *Aeschynomene fluminensis* Vell. were the most frequent (43.3 and 11.34%, respectively) plants encountered and were selected for the isolation of root endophytic fungi. These plants were collected in an area without mercury contamination to compare the effect of the metal on the structuring of the endophytic community. We collected five adult individuals with their root systems intact in each of the areas selected. The plant and soil samples were transported to the laboratory under refrigeration.

### Characterization of the cultivable community of endophytic fungi in roots

The healthy roots of *P*. *acuminatum* (P) and *A*. *fluminensis* (A) from were collected at sites with (+PHg and +AHg) and without mercury (-PHg and -AHg). Subsequently the roots were washed with neutral detergent (Ype® Neutral Detergent), rinsed superficially disinfected from standard protocol for isolation of endophytic fungi [13,16,20,29,43] with some modifications—immersion in ethanol 70% for 1min, and sodium hypochlorite 2.5% for 5min, followed by rinsing in sterile distilled water (5 times).

Twelve root fragments (~5mm length) of each sample were transferred to Petri dishes containing PDA medium (potato dextrose agar) supplemented with 30 μg. mL^-1^ of Hg^+2^ as HgCl_2_ and 100 μg. mL^-1^ of antibiotics (chloramphenicol, streptomycin and tetracycline). 120 fragments of roots from each plant species were plated onto each of ten Petri dishes for each plant species. The plates were maintained at 28°C and analyzed daily. The fungal strains emerging were purified and grouped into morphotypes [44].

The morphotypes were confirmed by microscopic characteristics observed on glass microscope slides obtained through microculture [45]. All the isolates were deposited and maintained under refrigeration in the collection of mercury resistant microorganisms from the Laboratory of Biotechnology and Microbial Ecology (LABEM), Federal University of Mato Grosso.

Total DNA from each monosporic isolate was extracted using DNA Purification Kit (Norgen Biotek Corp, Canada). The amplification profile ISSR-PCR was used to assist in morphotyping and species differentiation [46]. The morphotypes were submitted to molecular identification by sequencing the ITS regions using the primers ITS1 and ITS4 [47]. The amplicons were enzymatically purified (ExoSap-it, GE Healthcare) and sequenced by the Sanger method (BigDye Terminator Cycle Sequencing). The sequence consensus obtained from primers ITS1 and ITS4 was obtained on the MEGA 7 software [48] and compared with sequences obtained from the GenBank database through the nBLAST tool (http://www.ncbi.nlm.nih.gov). The sequences that shared 97% or more of similarities were identified as the same species [49]. The strains that could not be identified using the ITS region were submitted to the amplification and sequencing of the β-tubulin gene with the primers Bt2a and Bt2b [50] amplification program: denaturation at 95°C (2 min), 35 cycles of 94°C (45s), 50°C (45s) and 72°C (1 min), followed by final extension at 72°C for 10 min. The purification procedure, sequencing and analysis of the sequences were performed as described above. The sequences obtained were compared to the GenBank database through the nBLAST and xBLASTx tool. All sequences obtained in this work were deposited in GenBank with codes KX381110 to KX381202 for ITS and KY987116 to KY987119 for beta tubulin.

### Functional characterization of endophytic fungal strains and tolerance to Cd^2+^, Zn^2+^ and Pb^2+^

A strain of each species of endophytic fungus present in the evaluated communities was used to characterize functional traits: synthesis of indoleacetic acid (IAA) [22], siderophores production [51], hydrolytic enzymes secretion [52] and antibiosis against *Staphylococcus Saprophyticus* (ATCC 43867) and *Escherichia coli* (ATCC 25922) [53]. The tolerance of the strains to Cd^2+^, Zn^2+^ and Pb^2+^ was defined using Sabouraud culture medium, supplemented with CdSO_4_.8H_2_O (3mM), ZnSO_4_H_2_0 (20 mM), Pb(NO_3_)2 (10 mM) and control (0mM of heavy metal) [54].

### Growth of strains in the presence of mercury

The microorganisms were cultured in Sabouraud culture medium, supplemented with mercury (0 and 30 μg mL^-1^ of Hg^+2^, as HgCl_2_). The radial growth of the mycelium was evaluated every 24 hours until the stationary phase of growth. The mycelial growth rate (μ/day) was calculated during the exponential phase and its value was used to determine the tolerance index (TI) of the strains [29]. The strains with TI higher than 0.9 were selected for evaluation of plant growth promotion.

### Plant growth promotion in the presence of mercury

Strains selected according to the TI value (n = 32) were activated in PDA. The seeds of *A*. *fluminensis* were mechanically scarified, disinfested (immersion in ethanol 70% for 1min, sodium hypochlorite 2.5% for 5min and rinsed in sterile distilled water) and germinated in vases of 0.5 dm^3^ containing vermiculite and sand 1:1 (w:w).

After fifteen days, the seedlings were transferred to vases containing vermiculite and sand 1:1 (w:w). 1 mL of spore suspension (10^6^ conidia, mL^-1^) was inoculated onto the soil near from the roots. The root of seedling was engaged with two discs (1cm diameter) of medium containing mycelium for the not sporulating strains.

The plants were cultivated for 30 days in the absence of mercury (acclimation period), maintained at 70% field capacity of the substrate and fertilized weekly with 100% Hoagland solution [55].

After the acclimation period, the mercury doses were divided (60, 30 and 30 mg kg^-1^ of Hg^+2^ as HgCl_2_) with a 24 h interval between the applications until reaching the concentration of 120 mg kg^-1^ of Hg^+2^. Each treatment consisted of four plants. The control vases endophyte-free with mercury (C+Hg) and without mercury (C-Hg) were used for comparison.

The plants were collected 52 days after the transplant. The chlorophyll level was evaluated by portable chlorophyll gauge (SPAD-512, Minolta) at the time of collection. The plant height was determined with the support of a millimeter ruler. The roots were rinsed and dipped in EDTA (0.01M) for 30 minutes. The dry biomass of root and aerial part was determined after drying in a stove at 65°C until reaching a constant mass.

The percentage of growth promoter efficiency (GPE) was estimated to evaluate the effect of strain inoculation on plant growth [56].

### Data analysis

The relative frequency of root colonization by endophytic fungi was calculated [57]. Shannon-Weaver diversity index and the Hill series were estimated for each community [58,59]. The Venn diagram was constructed from species composition data (http://bioinformatics.psb.ugent.be/webtools/Venn).

The functional characterization of the strains was qualitatively evaluated and the results expressed in positive (+), for the strains that exhibited the functional traits, and negative (-), for those that did not exhibit the traits. The metal tolerance index (TI) was determined for the strains in media supplemented with heavy metals and calculated according to the following formula: Diameter mycelium treatment/Diameter of control mycelium with 7 days of inoculation. TI = 0 and TI.< 0 respectively indicate inhibition and sensitivity of the strains in the presence of heavy metals, values of TI ≥ 1 indicate resistance to metals [29].

The data were submitted to analysis of parametric variance (ANOVA), non-parametric (Kruskal Wallis) and Duncan test when pertinent. The Cluster analysis was performed using the Bray-Curtis distance based dissimilarity matrix using composite data of the four endophytic fungal communities (+PHg, -PHg, +AHg and -PHg). Taxa most abundant, which represented by 4 or more isolates, were used in non-metric multidimensional scaling (NMDS) using the Jaccard and Bray—Curtis.

All statistical analyses were performed by software R (version 3.2.2). For PERMANOVA we used the Adonis function of the Vegan package. Indicator species analysis was performed based on the IndVal index to identify microbial species that were significantly correlated to host or contamination using the “indval” function in the “labdsv” package.

## Results

### Physicochemical analysis of the soil and composition of the endophytic fungi community

The concentration of mercury at +Hg site (3.24 mg.kg^-1^) is above the limit of prevention (0.5 mg.kg^-1^) established by the National Council of the Environment of Brazil [60]. This place is contaminated by mercury (+Hg) at a concentration 1905 times greater than the areas not contaminated with mercury–Hg sites 1 and 2 (S1 Table).

In total, we measured 480 root fragments to determine the frequency of colonization (FC) (Table 1). The roots of *P*. *acuminatum* and *A*. *fluminense* growing in contaminated areas (+PHg and +AHg) had the highest mean values of FC and statistics different from the values of FC obtained from the roots of hosts collected in areas without contamination. The number of isolates, richness and diversity of endophytic fungi differ between contaminated environments (+PHg and +AHg) and uncontaminated environments (-PHg and -AHg) (Table 1).

**Table 1:** Frequency of colonization (FC), isolates, richness and Shannon-Wiener index (H') of endophytic fungi in plants. Strains obtained from *Polygonum acuminatum* and *Aeschynomene fluminensis* from contaminated (+PHg and +AHg) and uncontaminated areas (-PHg and -AHg).

Parameters		+PHg	+AHg	-PHg	-AHg
Isolates	Total	72	46	38	34
	Average*	6,1 ± 3,5^a^	3,8 ± 2,5^ab^	3,2 ± 2,0^b^	2,8 ± 2,4^b^
Richness	Total	31	27	18	18
	Average *	4,7 ± 2,4^a^	3,2 ± 1,8^ab^	2,7 ± 1,7^b^	2,3 ± 1,7^b^
H’	Total	3,03	3,10	2,47	2,77
	Average*	1,4 ± 0,5^a^	1,0 ± 0,6^ab^	0,8 ± 0,7^b^	0,6 ± 0,6^b^
FC (%)	Average **	54,1 ± 16,0^a^	48,3 ± 14,8^a^	26,5 ± 18,3^b^	30,8 ± 20,2^b^

Equal letters in the same row do not differ statistically by Duncan's test (* p = 0.01 and ** p = 0.001). The means are shown with ± SD

The mercury level has a statistically significant effect on richness (F: 8.054, P <0.01), diversity of the Simpson Index (F: 6.238, P <0.05) and abundance (F: 6.587; P <0.05). In contrast, the vegetation type had no effect on the richness (3.008; P = 0.09) diversity (F: 2,677, P = 0.10) and abundance (F: 2,866, P = 0.10).

**Fig 1:**
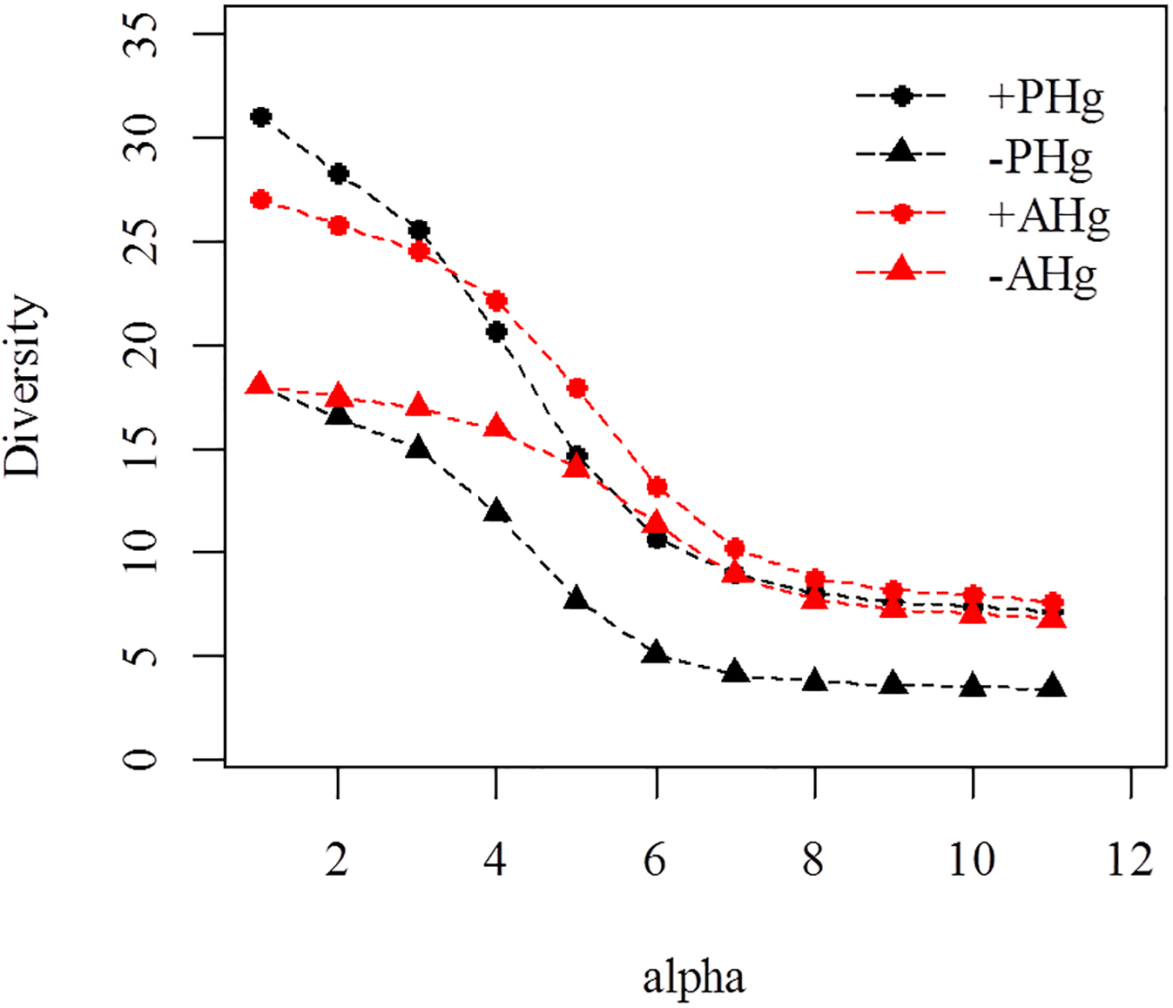
Endophytic diversity profiles. *Aeschynomene fluminensis* (red line) and *Polygonum acuminatum* (black line) of environments with (+PHg and +AHg) (●) and without (-PHg and -AHg) (▲) mercury, using the Hill Series. For the parameter *a* = 0, the diversity value is equal to the number of species in the sample. For the *a* tending to 1, the diversity value is equivalent to the Shannon index. For *a* = 2, the value equals the Simpson index.

The Hill series indicated higher values of richness, Shannon and Simpson indices for endophytic fungal communities colonizing hosts in the area impacted by mercury (+PHg and +AHg) (Fig 1). The equability was higher in these environments, represented by the slope of the curve of the diversity profiles.

We obtained 190 fungal isolates from root fragments that were distributed in two phyla, four classes, fifteen orders, twenty-seven families and thirty-five genera (S1 and S2 Figs; S2 Table). The phylum Ascomycota (96.84%), the class Sordariomycetes (51.05%), the order Pleoporales (37.89%), the family Glomerellaceae (11.58%) and the genre *Colletotrichum* (10.53%) were more abundant.

There is a predominance of Sordariomycetes for hosts collected in contaminated areas. Dothideomycetes are abundant in uncontaminated areas. The order of Pleosporales is proportional and larger in all analyzed communities (S1 and S2 Figs).

In relation with the families and genera, Glomerallaceae (19.70%) and *Colletotrichum* (18.31%) are abundant in +PHg, Lindgomycetaceae is the family most represented in -PHg (38.23%) and +AHg (13,79%). In -PHg, *Fusarium* was the most abundant genus (28.00%) and *Massariosphaeria* in +AHg (17.65%). The families Aspergiliaceae, Glomerallaceae and Trematosphaeriaceae correspond to 50.01% of the isolates obtained in -AHg roots, being in this community, *Colletotrichum* and *Falciformispora* (18,52% each) were the most abundant genera (S1 and S2 Figs).

In the case of the taxa, *Colletotrichum* sp. represented 7.37% of the isolates of the total isolates. The community of +PHg is represented mainly by *Trichoderma brevicompactum*, *Colletotrichum* sp. and *Diaporthe phaseolorum*, and together they represent 37.5% of the isolates. In -PHg, 28.5% of the isolates correspond to a single taxon, Lindgomycetaceae 1 (S2 Table).

For endophytic fungi of *A*. *fluminensis*, the abundant species are *Massariosphaeria* sp. (more abundant on root’s *A*. *fluminensis* from environment with mercury (+AHg, 13%)), and *Colletotrichum* sp. (more abundant on the host collected in areas without contamination (-AHg, 14.7%, S2 Table).

The molecular identification through sequencing of the ITS region was not possible for some strains (A11, A17, A18, A23, A24, A43, A59, A65, A73, P40, P74 and P87). The sequencing of the β-tubulin gene aided in taxonomic elucidation. It was possible to identify the P40 strain that belongs to the species *Microsphaeropsis arundinis* (S2 Table).

**Fig 2:**
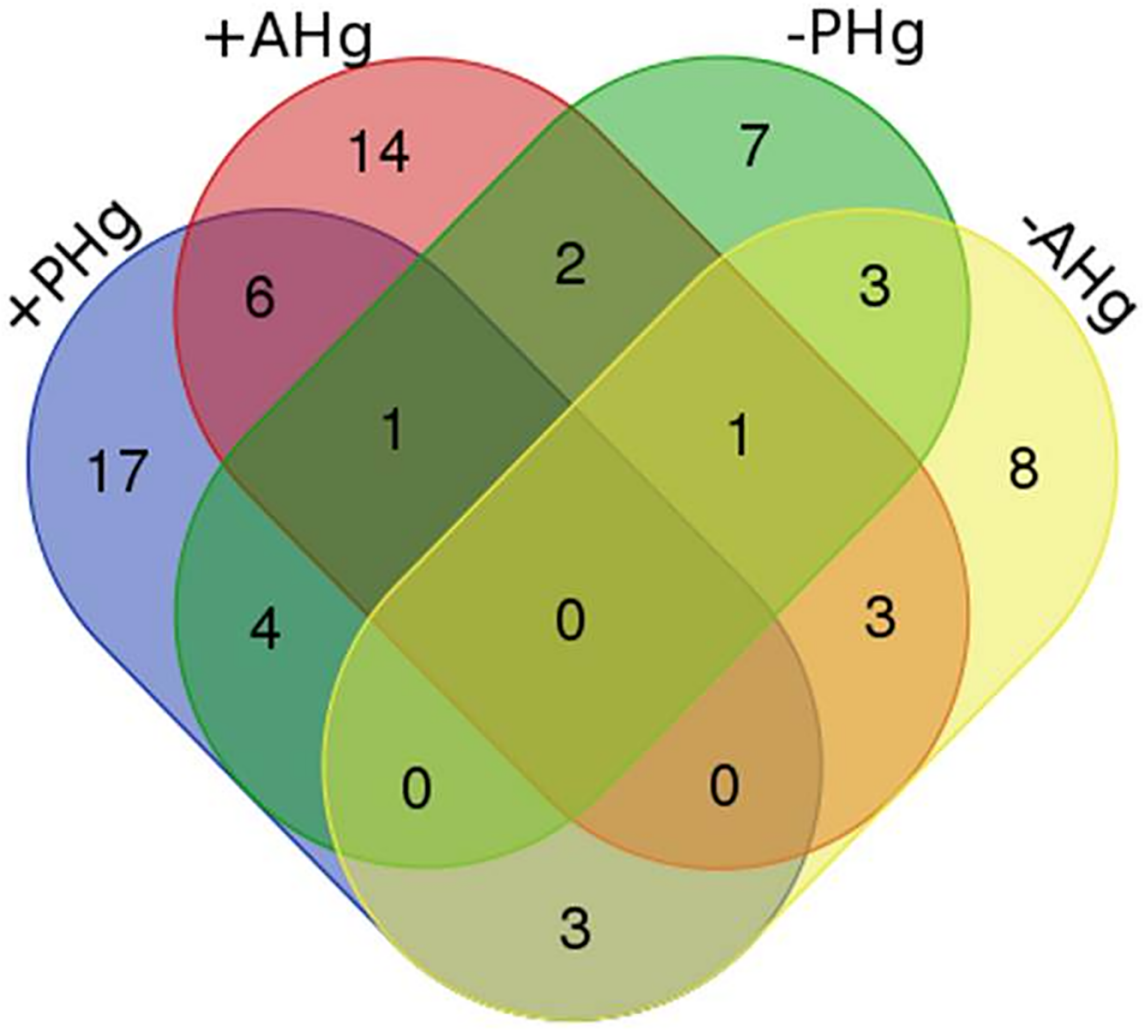
Venn diagram of endophytic fungi isolated. Strains obtained from *Polygonum acuminatum* and *Aeschynomene fluminensis* from contaminated (+PHg and +AHg) and uncontaminated areas (-PHg and -AHg).

The species *Colletotrichum gloeosporioides*, *D*. *phaseolorum*, *Lasiodiplodia pseudotheobromae* and *Phomopsis* sp.1 are typical taxons of *P acuminatum* independent of the environment, because they occur in the host independent of the mercury contamination (Fig 2, S2 Table). *Scedosporium apiospermum*, *Ceratobasidium* sp.2 and *Aspergillus* sp.2 are specific species to *A*. *fluminensis* independent of the type of environment (Fig 2, S2 Table).

Several species are host/environment specific, that is seventeen taxa only occur in *P*. *acuminatum* growing in areas with mercury and seven taxa were exclusive to hosts without contamination (Fig 2, S2 Table).

This pattern is also observed for *A*. *fluminensis*. There are fourteen species exclusive of +AHg and eight are restricted to the -AHg areas (Fig 2, S2 Table).

*Fusarium oxysporum*, Lindgomycetaceae 1 and *Falciformispora* sp.1 did not show specificity per host, colonizing roots of both plants, however, the species were only detected in uncontaminated environments. The opposite was verified for *Ceratobasidium* sp.2, *Ascochyta medicaginicola*, *Falciformispora* sp.2, *Trichoderma brevicompactum*, *Pestalotiopsis* sp., *Microsphaeropsis arundinis*, its species restricted to contaminated environments, independent of the host (Fig 2; S2 Table).

*D*. *phaseolorum* is the indicator species for *P*. *acuminatum* considering the type of host (indicator value: 0.2917, P = 0.011). Lindgomycetaceae 1 (indicator value: 0.3750, P = 0.004) and *F*. *oxysporum* (indicator value: 0.2500, P = 0.023) are indicators of uncontaminated areas, while *Massariosphaeria* sp (indicator value: 0.3053, P = 0.021) and *T*. *brevicompactum* (indicator value: 0.2500, P = 0.022) are species indicative of areas contaminated by mercury.

The communities of endophytic fungi from plants from places without mercury (-PHg and -AHg) are similar in relation to species composition, according to the grouping analysis constructed from the Jaccard index (S3 Fig).

**Fig 3:**
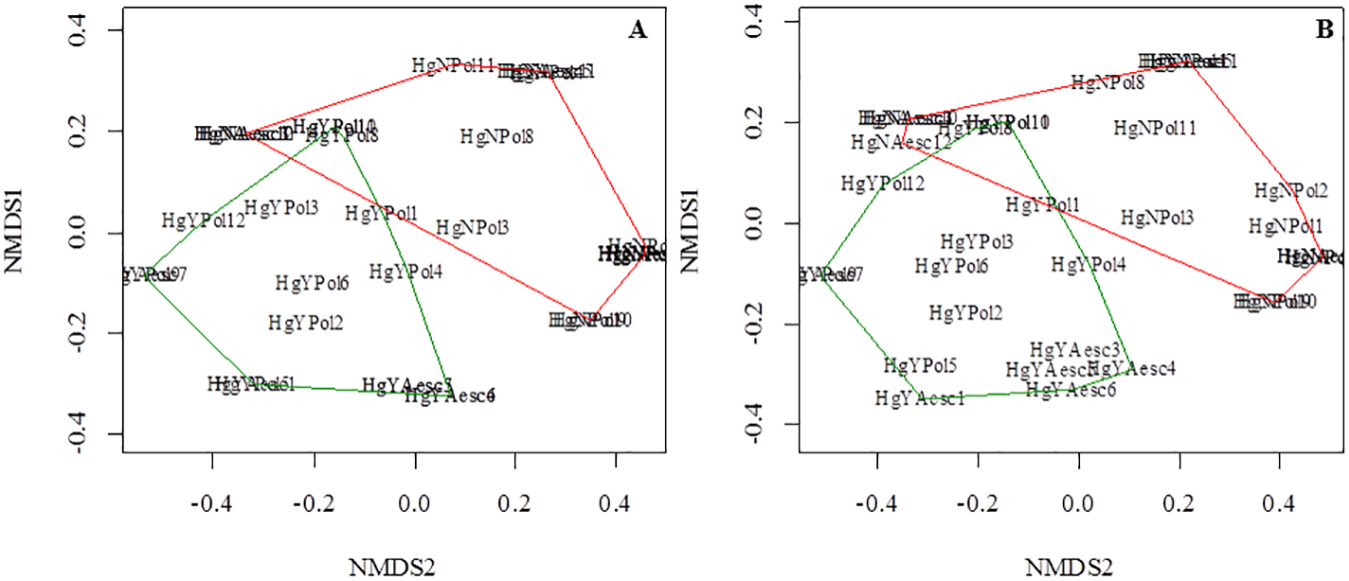
**Non Metric Scheduling (NMDS) calculated from the Jaccard (A) and Bray-Curtis (B) distances for host isolates from mercury contaminated and uncontaminated areas**. The green and red polygons show the grouping between isolates from the contaminated and uncontaminated areas.

The most abundant taxa in terms of isolate number are influenced by mercury. The results were obtained from not metric multidimensional scaling (NMDS), where the grouping of species as a function of presence (green line) and absence of mercury (red line) was observed, both for the composition distance of Jaccard (Fig 3A) as for abundance Bray-Curtis (Fig 3B). The two groups formed are statistically different for both distances according to PERMANOVA (R^2^ = 0.16, P = 000.1). There was not statistically significant grouping according to the type of host (PERMANOVA, R^2^ = 0.04 and P = 0.18).

### Functional characterization of endophytic fungal communities and tolerance to Cd^2+^, Zn^2+^ and Pb^2+^

The functional profile of the endophytic fungal community was determined by the qualitative analysis of important traits in the promoter of plant growth.

The percentage of strains from contaminated and functional trait producing areas is bigger than those isolated strains from hosts growing in uncontaminated areas, except for lipase synthesis and indoleacetic acid (IAA) (Table 2).

**Table 2:** Functional traits and metal tolerance (Cd^2+^, Zn^2+^ and Pb^2+^) expressed in percentages of endophytic fungi isolated. Strains obtained from *Polygonum acuminatum* and *Aeschynomene fluminensis* from contaminated (+PHg and +AHg) and uncontaminated areas (-PHg and -AHg).

Functional traits (%)		+PHg	+AHg	-PHg	-AHg
Enzymes	Protease	26.9	38.7	22.2	27.8
	Amylase	73.1	96.8	83.3	66.7
	Cellulase	38.5	29.0	33.3	33.3
	Ligninase	73.0	48.4	38.9	61.1
	Lipase	23.1	29.0	27.8	33.3
Antibiosis	*S*. *saprophyticus*	7.7	16.1	16.7	5.6
	*E*. *coli*	15.4	3.2	11.1	0.0
Pb^+2^	TI > 1	19.2	3.2	16.7	5.6
	TI = 1	11.5	6.4	16.7	11.1
	TI < 1	69.2	83.9	61.1	83.3
	TI = 0	0.0	6.4	5.6	0.0
Zn^+2^	TI > 1	0.0	3.2	0.0	0.0
	TI = 1	0.0	3.2	0.0	5.6
	TI < 1	73.1	48.4	72.2	72.2
	TI = 0	26.9	45.2	27.0	22.2
Cd^+2^	TI > 1	0.0	0.0	0.0	5.6
	TI = 1	0.0	3.2	0.0	0.0
	TI < 1	38.5	38.7	50.0	33.3
	TI = 0	61.5	58.1	50.0	61.1
Siderophore		35.5	46.1	22.2	33.3
Indoleacetic acid (IAA)		71.0	50.0	72.2	55.6
**Total of isolates**		**31**	**26**	**18**	**18**

TI = Tolerance index

The number of siderophore-producing strains was higher in isolates from +AHg and +PHg (46.1 and 35.5% of isolates) compared to -AHg and -PHg (33.3 and 22.2%). The production of IAA was higher in isolated strains of -PHg (72.2% of the isolates, Table 2).

The number of isolate-producers of hydrolytic enzymes was higher for communities in the contaminated area: amylase and protease (96.8% and 38.7%) in +PHg. and lignase and cellulase in +AHg (73.0 and 38.5%). Only for lipase that were observed a higher strain number for -AHg isolates (33.3%) (Table 2). Two strains isolated from the contaminated area, *Aspergillus japonicus* A32 and *Emericellopsis* sp P54 were positive for the five enzymes evaluated (S3 Table).

The strains obtained from the four communities were able to inhibit the growth of Gram-negative and Gram-positive strains of bacteria (S3 Table). Of these strains, *Clonostachys rhizophaga* P89, *Aspergillus* sp.1 A25, Glomeralleceae A43, *Westerdykella* sp.1 P71 showed activity for both pathogenic bacterias (S3 Table).

All isolates of *A*. *fluminensis* from both environments were resistant (TI > 1) or sensitive (TI < 1) to Pb^2+^ (Table 2, S3 Table). Interesting, about 19.2% of the +AHg isolates were stimulated by the addition of Pb^2+^ in the culture medium (TI > 1). *Aspergillus* sp.1 A25 is tolerant to this metal (TI = 2.69). The majority of the strains were sensitive or inhibited by Zn^2+^. Only *Bipolaris setariae* P4 and *Phomopsis* sp. P49 were resistant to Zn^2+^ (TI = 1.06 and 1.00 respectively, S3 Table).

The Cd^2+^ was the most toxic metal to endophytic fungal strains, especially those obtained from -PHg. This metal was able to reduce or inhibit mycelial growth, except for the fungi *Falciformispora* sp.3 A76 (TI = 1.20) and *Trichoderma harzianum* P39 (TI = 1) which were shown to be stimulated or resistant to cadmium, respectively (Table 2; S3 Table).

### Growth of strains in the presence of mercury

Mercury tolerance was determined by the mycelial growth rate in 0 and 30μg mL^-1^ of Hg^2+^. According to the tolerance index, 80% of the evaluated strains were sensitive to Hg^2+^ (TI < 1) and only Chaetosphaeriales A24 was completely inhibited by metal (TI = 0) (S2 Table). In addition, different resistant strains were observed in all analyzed communities (15.71%), especially in non-mercury contaminated hosts (S2 Table). Among the resistant strains, *Cochliobolus* sp. P86 (TI = 2.1), *Massariosphaeria* sp. A19 and Fungal A17 (TI = 1.5 each), Lindgomycetaceae 1 P87 (TI = 1.3) and *Cladosporium uredinicola* A72 (TI = 1.2) were the most resistant strains to the metal (Table 3, S2 Table). The strains with TI higher than 0.9 represented about 42% of the endophytic fungi evaluated, that is equivalent to 32 species. These strains were selected for inoculation with *A*. *fluminensis* in a condition of contamination with Hg^+2^ (Table 3).

**Table 3:** Growth promotion of *Aeschynomene fluminensis* inoculated with endophytic fungal strains cultivated under mercury contamination. Strains obtained from *Polygonum acuminatum* and *Aeschynomene fluminensis* from contaminated (+PHg and +AHg) and uncontaminated area (-PHg and -AHg).

Sample	Strains	TI (Hg^+2^)	Dry biomass ^(1)^		Plant height ^(1)^		Chlorophyll ^(3)^ (SPAD)
			(g. plant ^-1^)	GEF^(2)^	cm	GPE^(2)^	
*-*AHg	*Aspergillus* sp.1 A51	0.9	0.505 ± 0.108 ^a^	363.30	19.73 ± 3.94 ^a-d^	90.08	28.30 ± 2.99 ^ab^
*+*AHg	*Massariosphaeria* sp. A19	1.5	0.455 ± 0.063 ^ab^	317.66	21.68 ± 1.85 ^ab^	108.86	31.63 ± 4.80 ^a^
*+*AHg	Sardoriomycetes A18	0.9	0.447 ± 0.091 ^ab^	309.86	24.28 ± 2.13 ^a^	133.91	20.23 ± 0.90 ^a-d^
*-*PHg	Lindgomycetaceae 1 P87	1.3	0.422± 0.087 ^ab^	286.70	21.18 ± 1.86 ^a-c^	104.05	26.93 ± 3.14 ^ab^
*-*AHg	*Cladosporium uredinicola* A72	1.2	0.382 ± 0.070 ^bc^	250.00	18.65 ± 2.63 ^a-f^	79.67	24.60 ± 4.10 ^a-d^
*+*AHg	*Ascochyta medicaginicola* A9	0.9	0.366 ± 0.047 ^bc^	235.32	18.95 ± 3.43 ^a-e^	82.56	25.50 ± 3.68 ^a-c^
*+*AHg	*Aspergillus* sp.2 A31	0.9	0.300 ± 0.032 ^c^	175.23	17.40 ± 1.13 ^b-g^	67.63	30.76 ± 5.98 ^ab^
*+*AHg	*Scedosporium boydii* A38	0.9	0.193 ± 0.041 ^de^	77.06	11.73 ± 4.93 ^g-k^	13.01	14.83 ± 2.32 ^a-d^
*+*PHg	*Colletotrichum* sp. P42	1	0.189 ± 0.07 ^d^	73.39	15.83 ± 4.33 ^b-h^	52.5	21.75 ± 4.01 ^a-d^
*-*AHg	*Phlebiopsis* sp. A75	1	0.161 ± 0.030 ^de^	47.71	11.88 ± 3.49 ^g-k^	14.45	20.70 ± 0.33 ^a-d^
*-*AHg	*Westerdykella* sp.2 A47	1.1	0.153 ± 0.036 ^d-f^	40.37	10.35 ± 1.61 ^h-k^	-0.29	16.50 ± 1.53 ^a-d^
*+*PHg	*Colletotrichum gloeosporioides* P24	1	0.152 ± 0.047 ^d-f^	39.68	13.38 ± 2.44 ^e-k^	28.9	20.85 ± 4.86 ^a-d^
*+*PHg	*Curvularia geniculata* P1	0.9	0.152 ± 0.055 ^d-f^	39.45	10.73 ± 3.16 ^h-k^	3.37	12.76 ± 1.98 ^a-d^
*+*PHg	*Clonostachys rogersoniana* P62	1	0.142 ± 0.036 ^d-f^	29.82	9.98 ± 2.50 ^h-k^	-3.85	20.33 ± 4.13 ^a-d^
*+*PHg	*Penicillium oxalicum* P32	1.1	0.139 ± 0.024 ^d-f^	27.29	12.48 ± 3.19 ^f-k^	20.23	20.70 ± 0.99 ^a-d^
*+*PHg	*Phoma* sp 2 P67	0.9	0.136 ± 0.041 ^d-f^	24.31	10.63 ± 1.11 ^h-k^	2.41	17.03 ± 2.86 ^a-d^
*-*PHg	*Westerdykella* sp.1 P71	1	0.131 ± 0.033 ^d-f^	20.18	10.55 ± 2.25 ^h-k^	1.64	16.03 ± 4.70 ^a-d^
*+*PHg	*Cochliobolus geniculatus* P59	1	0.131 ± 0.065 ^d-f^	19.72	9.55 ± 3.95 ^h-k^	-8	7.66 ± 0.17 ^a-d^
*-*PHg	*Diaporthe miriciae* P96	0.9	0.129 ± 0.024 ^d-f^	18.58	10.50 ± 2.97 ^h-k^	1.16	21.33 ± 2.31 ^a-d^
*-*AHg	*Falciformispora* sp.1 A49	1	0.129 ± 0.050 ^d-f^	18.58	12.85 ± 4.65 ^e-k^	23.8	21.73 ± 4.99 ^a-d^
*-*PHg	*Hongkongmyces pedis* P107	0.9	0.127 ± 0.057 ^d-f^	16.06	11.63 ± 3.16 ^g-k^	12.04	19.76 ± 3.41 ^a-d^
*-*PHg	*Dokmaia* sp. P113	0.9	0.119 ± 0.014 ^d-f^	9.40	9.73 ± 0.71 ^h-k^	-6.26	8.56 ± 1.86 ^a-d^
*-*PHg	*Cochliobolus* sp. P86	2.1	0.119 ± 0.065 ^d-f^	9.40	10.05 ± 1.05 ^h-k^	-3.18	21.35 ± 2.27 ^a-d^
*+*AHg	Ascomycota A17	1.5	0.113 ± 0.008 ^d-f^	3.90	11.90 ± 0.62 ^g-k^	14.64	12.05 ± 2.57 ^a-d^
*+*AHg	*Microsphaeropsis arundinis* A36	1	0.108 ± 0.032 ^d-f^	-0.69	9.93 ± 2.22 ^h-k^	-4.34	7.65 ± 2.21 ^a-d^
*-*AHg	*Fusarium oxysporum* A64	1	0.107 ± 0.050 ^d-f^	-1.83	11.78 ± 1.79 ^g-k^	13.49	3.46 ± 0.84 ^d^
*-*AHg	*Penicillium janthinellum* A56	1	0.105 ± 0.037 ^d-f^	-3.44	12.78 ± 2.40 ^e-k^	23.12	7.10 ± 0.83 ^a-d^
*-*PHg	*Acrocalymma vagum* P18	0.9	0.105 ± 0.070 ^d-f^	-4.13	11.38 ± 3.88 ^g-k^	9.63	7.26 ± 0.77 ^a-d^
*+*AHg	*Scedosporium apiospermum* A42	0.9	0.086 ± 0.064 ^d-f^	-20.87	7.43 ± 2.16 ^jk^	-28.42	16.70 ± 2.14 ^a-d^
*-*PHg	*Clonostachys rhizophaga* P89	1	0.078 ± 0.020 ^d-f^	-28.13	10.57 ± 2.13 ^h-k^	1.83	6.20 ± 0.67 ^b-d^
*-*PHg	*Trichoderma brevicompactum* P35	1	0.074 ± 0.03 ^ef^	-32.11	6.9 ± 0.95 ^k^	-12.81	13.35 ± 2.82 ^a-d^
*+*AHg	*Aspergillus japonicus* A32	1	0.070 ± 0.020 ^f^	-35.89	9.05 ± 5.07 ^i-k^	-33.53	4.15 ± 0.78 ^b-d^
** **	C—Hg	-	0.179 ± 0.071 ^de^	64.22	13.78 ± 5.05 ^d-j^	32.76	20.47 ± 5.99 ^a-d^
** **	C + Hg	-	0.109 ± 0.034 ^d-f^	-	10.38 ± 3.37 ^h-k^	-	6.13 ± 2.99 ^b-d^

C-Hg No-inoculated plants without Hg and C+Hg No-inoculated plants with Hg. Letters in the same column do not differ statistically (^1^ Duncan test, ^2^ Growth promotion efficacy (%) and ^3^ Kruskal Wallis)

### Host growth promotion tests in the presence of mercury

*A*. *fluminensis* is a sensitive species to 120 mg kg^-1^ of mercury. The addition of mercury resulted in a reduction in the dry mass and height of non-inoculated plants by approximately 40% and 24%, respectively (Table 3). The use of mercury tolerant fungal strains positively influenced the growth of *A*. *fluminensis* in mercury contamination conditions (Table 3).

According to growth promotion efficiency (GPE), twenty-four strains provided an increase in the host's dry biomass, with GPE values ranging from 363.30 to 3.90% (Table 3). Despite increasing dry biomass (GPE between 1.16 and 133.91%), five of these strains reduced total length in relation to non-inoculated plants (GPE ranging from -0.29 to -8.00). Surprisingly, nine strains promoted the growth of *A*. *fluminensis* with parameters of GPE (dry biomass and length) greater than those obtained for non-inoculated plants and those without addition of mercury to the substrate (C-Hg). These microbes may be promising microorganisms to enhance plant growth in bioremediation programs (Table 3). Most of the promising strains were isolated from *A*. *fluminensis* (7) from the contaminated site (6). Only Lindgomycetaceae 1 P87 and *Colletotrichum* sp. P42 were isolated from *P*. *acuminatum* (Table 3).

Eight strains (five of *A*. *fluminensis* and three of *P*.*acuminatum*) inhibited the accumulation of dry biomass in *A*. *fluminensis* (GPE ranging from -0.69 to -35.89%), causing reduction (GPE between -4.34 and -33.53) or increase in total length of plants (GPE between 1.83 and 23.12). Plant inoculation with *T*. *brevicompactum* P35 resulted in the lowest GPE values of dry biomass (-35.89) and length (-33.53) inhibiting the growth of *A*. *fluminensis* (Table 3).

Mercury contamination affects chlorophyll synthesis in *A*. *fluminensis* verified by the chlorophyll index (SPAD). The addition of heavy metal resulted in a lower SPAD index (6.13 ± 2.99) compared to the measurements obtained from plants growing on a substrate without mercury (20.47 ± 5.99) (Table 3). There was a positive correlation (with significance at 0.01) between the SPAD indices and the values of dry biomass (Pearson Correlation 0.721) and the plant length (Pearson Correlation 0.659) indicating that fungi that promoted plant growth provided higher SPAD indices in the leaves of the host. The inoculation of the host with endophytic fungi contains the effect of mercury on chlorophyll synthesis, 93.75% of the strains improved chlorophyll index in relation to the uninoculated plants (C + Hg). Of this amount, 42.33% showed chlorophyll values higher than those recorded in non-inoculated plants and without mercury (C-Hg).

The highest SPAD index was obtained in plants inoculated with *Massariosphaeria* sp. A19 (31.63 ± 4.80) (Table 3). Only inoculation with *F*. *oxysporum* P80 (isolated of *P*. *acuminatum*) and *A*. *japonicus* A32 (isolated of *A*.*fluminensis*) showed a deleterious effect on the concentration of chlorophyll in the host (Table 3).

## Discussion

The history of gold mining in the city of Poconé began with large scale exploration in the 1980s, resulting in one of the biggest anthropogenic impacts on the Pantanal: mercury contamination in bodies of water [4], sediments [3] and fauna [5–7].

Plants have different strategies to colonize soils containing high concentrations of heavy metals and only resistant species can occupy this niche [61]. These strategies include the activation of genes that encode multiple enzymatic pathways and are related to the process of metal exclusion of the plant/root or mechanisms of tolerances that allow the accumulation of the metal inside the plant [62].

Fungi tolerant to heavy metals may influence plant occupation in old mining areas. The inoculation of the host with resistant strains may result in the promotion of plant growth in metal contaminated environments.

In this work, we observed that *A*. *fluminensis* and *P*. *acuminatum* are abundant in soils of wetland areas contaminated by mercury. Our results demonstrate that the fungal community structure of the roots of these hosts have important functions for the colonization of contaminated environments.

### Influence of mercury contamination on the structuring of endophytic fungi communities

The community of endophytic fungi in areas contaminated by heavy metals has been evaluated to obtain tolerant strains for use in bioremediation programs [15,16,20,63,64]. However, the effects of toxic metals on the structure and function of the endophytic fungal communities have been poorly investigated. Therefore, our approach intends to develop an understanding of the effects of mercury on the root endophytic fungal communities of plants and the potential to affect host adaptability under conditions of environmental contamination.

The measurements of colonization of endophytes in fragments of plant tissues may indicate the dynamics and extent of colonization of the host [57]. It was verified that environmental contamination with mercury is accompanied by higher colonization frequencies in the evaluated hosts (Table 1). These values have a direct relationship with the number of isolates, richness and diversity of root endophytic fungi obtained in hosts collected in contaminated areas by mercury (Table 1). Possibly, the existence of adaptive mechanisms in response to the period of exposure to mercury allows the survival and selection of endophytic fungi in stressed environments.

Several factors affect the composition and structure of endophytic fungi, such as salinity [29], soil management [65], climate [66] and host [67]. In the same way, the presence of contaminants, such as heavy metals, are important parameters in structuring the fungal community [12,17,68].

Endophytic fungi tolerant to heavy metals are often obtained from hosts of contaminated areas [16,20,63,69], and few researches have evaluated the pattern of fungal endophytic diversity on the effect of these metals, especially mercury. The contamination with zinc and cadmium does not influence the diversity of endophytic fungi [12]. In contrast, soil contamination with cadmium, lead and zinc increased the diversity of endophytic fungi [17], while the diversity of mycorrhizal fungi was reduced in the presence of heavy metals [70,71].

In our study, the composition and structure of the most abundant taxa of endophytic fungi were influenced by contamination with mercury and not by host identity (Fig 3). Although the host genotype is an important factor for structuring fungal communities [72], changes in the communities of endomycorrhizal [68] and ectomycorrhizal fungi [11,12] have been attributed to the concentrations of heavy metals.

The Ascomycota phylum and the Pleosporales order were dominant in the composition of the evaluated communities (S1 and S3 Figs). Ascomycotas are frequent and represent a good portion of isolated strains of different hosts [17,29,57,73,74]. The most abundant order in this research Pleosporales is represented by functionally versatile species, with a broad phenotypic plasticity and capable of adaptation in a variety of environments [75].

Specific strains of a plant and environment (Fig 2) reveal a specificity of the endophytic fungi with its host, as observed for endophytic fungi of arboreal species [74], ectomycorrhizal [76] and arbuscular mycorrhizal fungi [77]. Some fungal species were identified only in host roots growing in the presence (*Ceratobasidium* sp.2, *Phoma* sp.1, *Falciformispora* sp.2, *T*. *brevicompactum*, *Pestalotiopsis* sp.) or in absence (*F*. *oxysporum*, Lindgomycetaceae 1 and *Falciformispora* sp. 1) of mercury. Other species showed specificity with the type of host analyzed, such as *C*. *gloesporioides*, *D*. *phaseolorum*, *L*. *pseudotheobromae* and *Phomopsis* sp. in *P*. *acuminatum* and *S*. *apiospermum*, *Ceratobasidium* sp.2 and *Aspergillus* sp.1 in *A*. *fluminensis*.

In our research, *Colletotrichum* was the most abundant genus and together with *Phoma*, *Fusarium*, *Diaporthes*, *Phomopsis*, *Aspergillus* and *Trichoderma* represented more than 30% of the total isolates. These genera are frequent for endophytic fungi from areas contaminated with heavy metals [17,19,20,57]. Although usually considered a plant pathogen [78], species of genus *Colletotrichum* are frequently endophytic in foilar tissues [20,65,73,79] and less frequently in root tissues [79]. However, in our study isolates of *Colletotrichum* were abundant, possibly because these strains are important to alleviate the stress caused by mercury. An abundant strain of *Colletotrichum* (P42) promoted the growth of *A*. *fluminensis* grown in the presence of mercury. In addition, isolates of *Massariosphaeria*, *Falciformispora* and *Hongkongmyces* were abundant genera in *A*. *fluminensis* in the contaminated area, and as endophytes of plants in heavy metal contaminated areas. The isolation of these fungi from these less examined plants may be an indication these fungi may be used as an environmental biotechnological tool, expanding the spectrum of microbes with potential for use in bioremediation processes.

Although many endophytic *Fusarium* isolates are representative of host communities of areas contaminated with heavy metals [17,20,57,64], the species *F*. *oxysporum* colonizes only plants from places that are free from contamination with heavy metals [29,45,73,80,81]. Our results confirm this observation, since isolates of *F*. *oxysporum* were indicative of Hg^+2^ free areas. In contrast, *Massariosphaeria* sp and *T*. *brevicompactum* were indicative of contaminated areas. *Massariosphaeria* has not yet been reported as endophytic, however the genus has been reported for humid environments, being isolated from samples of wood [82], herbaceous substrate [83] and decomposing *Phragmites australis* tissues [84,85], suggesting that the genus possesses an important role in nutrient cycling in humid areas. Our work corroborates this, because in addition to mercury resistance (S2 Table), *Massariophaeria* strains secrete amylase and lignase (S3 Table). In contrast, *Trichoderma* is consistantly associated with contaminated environments with heavy metals and can neutralize the effects of heavy metals on hosts [16,22]. *T*. *brevicompactum* is a rare new species that was first isolated in 2004 [86]. In addition to Hg^+2^ tolerance, the species is known for its antifungal properties [87].

Species of the genus *Hongkongmyces* were isolated in both environments and plants. The occurrence of endophytic fungi in both areas suggests a greater adaptive plasticity whose mechanisms of resistance to mercury are yet to be evaluated. *Hongkongmyces* has been described as a human pathogen, but the genus is embedded in the Lindgomycetaceae family, in which the isolates are unique to submerged plant stems from moist environments, as in our study [88]. The taxon most abundant in–PHg was classified as Lindgomycetaceae 1. Species of Lindgomycetaceae have hyper-diverse ITS regions [88], with some phylogenetic problems leading to polyphyletic species [89]. These characteristics make it difficult to identify them exclusively by homology from ITS sequences.

### Functional characterization of the endophytic fungal community

Endophyte-plant interactions contribute to the functioning of terrestrial ecosystems, modulating various plant ecological features such as productivity, colonization and plant diversity with direct influence on the nutrient cycle [27,80]. The species specificity demonstrated by some endophytic fungal strains suggests the existence of distinct functional roles.

The functional traits evaluated in the present research comprise three main functions performed by the plant microbiome: 1) participation of microorganisms in processes that relieve the stress of their hosts; 2) host defense against biotic aggressions; and 3) increased plant nutrition through the supply of nutrients [90].

The proportion of fungal strains that produce siderophores, amylases, proteases, ligninases, cellulases and exhibit antibiosis against bacteria was greater for the plant isolates from the contaminated areas (Table 2). This suggests that these traits are important in the multitrophic interactions between plants and microorganisms, which are mainly driven by environmental conditions and evolutionary selection processes [91,92].

Two strains (*A*. *japonicus* A32 and *Emericelopsis* sp P54) synthesized all the evaluated hydrolytic enzymes (S3 Table). The synthesis of hydrolytic enzymes by the endophytes enable the successful colonization of host tissues [26], as well as participating in nutrient cycling [25] and antagonism processes [93].

*Aspergillus* sp.2 A25, *C*. *Rhizophaga* P89, Glomerallaceae A43 and *Westerdykella* sp.1 P71 can protect plants against phytopathogenic bacteria (S3 Table), given antibiosis activity against Gram+ and Gram- strains [72].

The endophytic *Westerdykella* sp.1 P71 produced siderophores and IAA. These two functional traits are important for host growth in environments impacted by heavy metals, because siderophores support iron acquisition and chelation of harmful compounds [35] and IAA regulates plant growth and development [19,22].

Heavy metal tolerance indices vary among 93 endophytic fungal strains, including the strains of the same species, such as *Falciformispora* sp.1 isolated from an uncontaminated area. While the P92 strain is resistant to Pb^+2^ and sensitive to other metals (Zn^+2^ and Cd^+2^), A49 showed sensitivity only for Pb^+2^ and did not grow in the presence of Zn^+2^ and Pb^+2^ (S3 Table). Results obtained by Li [57] Shen [15], An [64] and Li [94] show that heavy metal tolerance often varies between strains of the same species. This plasticity seems to equate with multigenic control of metal resistance [95]. Additionally, differences in tolerance between strains reflect different strategies or adaptation mechanisms developed by the fungi, such as permeability barriers, metal and intracellular sequestration, efflux pumps, enzymatic detoxification and metal speciation [96].

Heavy metal tolerant strains were found both in the community obtained in contaminated environments and those isolated from hosts collected in mercury free environments (S3 Table). Among the tolerant strains, *Aspergillus* sp.2 A25, *B*. *setariae* P4 and *Falciformispora* sp.3 A76 were stimulated by metals and presented the highest tolerance index for Pb^+2^ (2.69), Zn^+2^ (1.06) and Cd^+2^ (1.2) as well as *Peyronellae* (J934 and J97) presented higher growth in the culture media supplemented with Pb^+2^ and Zn^+2^ [15]. Efficient physiological and molecular mechanisms allow organisms to carry out extracellular or intracellular detoxification processes [69] and thus ensures resistance to the metal.

At the concentrations tested, the cadmium was the most toxic metal, likely because of its ability to kill spores and cause DNA damage [97,98].

We found 33 endophytic strains that grew in all evaluated heavy metals (S3 Table). The occurrence endophytic fungal strains tolerant to heavy metals may be an important attribute for plant survival in areas impacted by heavy metals [99]. Therefore, endophytic fungi are reported to increase plant biomass in systems contaminated with toxic metals [15,54,57,63,64,69].

### Growth of strains in the presence of mercury

There are no records of endophytic fungi with the capacity to promote the mercury remediation. However, strains from both environments (-Hg and +Hg) were tolerant to Hg^+2^ and other metals evaluated (Pb^+2^, Zn^+2^ and Cd^+2^), thus tolerance capacity did not depend on the origin of the endophytic fungus [15].

It was found that 11 strains distributed in eight genera, from both hosts of contaminated area (6) and non-contaminated area (5) are stimulated (TI > 1) by Hg^+2^ (Table 3, S2 Table). Endophytes with mercury tolerance can be used to facilitate phytoremediation. Mercury is a non-essential metal [100], but it serves in *Cochliobolus* sp. P86 some unknown physiological function, which influenced mycelial growth. The strain P86 is stimulated by Hg^+2^ and its growth was twice as rapid in the presence of the metal (TI = 2.1). The tolerance of *Cochliobolus* to heavy metals has been poorly investigated, but it is known that the genus often has tolerance to xenobiotics, such as octyltin [101]. Resistant or heavy metal tolerant fungi have different physiological and biochemical mechanisms controlled by different resistance genes that act intracellularly and extracellularly to neutralize their toxicity or even compartmentalize the heavy metal [69].

The mercury resistance mechanisms of fungi have not yet been fully elucidated. Unlike bacteria that are able to transform and detoxify certain forms of mercury, using processes that evolve the production of enzymes, mediated by *mer* operon [102]. For soil fungi, such as strains of *Aspergillus niger* and *Cladosporium cladosporioides*, biovolatilization seems to be the main mechanism of resistance to Hg^+2^. In *T*. *harzianum* the expression of genes for the coding of hydrophobins is related to the process of fixation of mercury inside the fungal cell [103]. *A*. *niger* strains KRP2 and *A*. *flavus* KRP1 demonstrated growth capacity in mercury, but with lower TI (0.807 and 0.793, respectively) compared to the values we obtained for most of our strains [100].

*Massariosphaeria* sp and Lindgomycetaceae 1 were dominant and certain strains (A19 and P87) exhibited high TI values (1.5 and 1.3, respectively). There seems to be a relationship that the dominant endophytic fungi in heavy metal contaminated areas are those that present better tolerance to the contaminant [94]. These endophytes, thus, may be important for the establishment of the host in contaminated sites [94]. Furthermore, the strains A19 and P87 have dark pigmented mycelium, probably due to the concentration of melanin in the cell wall of the fungi. Melanized fungi are generally more resistant to stress conditions, and their survival being increased by melanin's neutralizing effect of oxidants generated by environmental stress; also, the melanin prevents the entry of heavy metal into the fungal cell interior [104].

The endophytic fungi with increased growth in the presence of mercury (TI > 1), as the strains P86, A19, A17, P87, A72, A47 and P32, have the potential to improve phytoremediation of the soils contaminated with mercury. However, in order to evaluate the highest possible number of strains for the ability to promote host growth under Hg^+2^ contamination conditions, we adopted TI ≥ 0.9 as a criterion for selecting strains (Table 3; S2 Table). This tolerance value is above the values recorded for mercury tolerant soil strains such as *A*. *niger* KRP2 and *A*. *flavus* KRP1 [100].

### Effect of endophytic fungi on plant growth in contaminated substrates

It is documented that plant species success in areas with biotic and abiotic stresses is often modulated by symbiotic associations with endophytes, a phenomenon referred to as “habitat adapted symbiosis” [21,105]. For this reason, isolates of endophytic fungi with growth capacity in Hg^+2^ were selected for growth promotion tests in the presence of the metal using *A*. *fluminensis* as host (Table 3).

All communities presented mercury resistant strains, including those that came from the uncontaminated area (Table 3), confirming the theory that isolates from contaminated and uncontaminated areas had metal tolerance [15,106]. In this way, other mechanisms may be involved in the tolerance process and not only the environment/habitat.

*A*. *fluminensis* has been shown to be sensitive to mercury and dependent on the mutualistic association to grow in contaminated soils, as well as reported for *C*. *barbinervis* [18].

In this research, several strains promoted host growth under mercury contamination. Nine endophytes (A51, A19, A18, P87, A72, A9, A31, A38 and P42) stimulated host growth above uninoculated and non-mercury treatments and were therefore promising microorganisms for studies of endophyte-facilitated phytoremediation (Table 3). IAA production by *Aspergillus* strains (A51 and A31) and *Massariosphaeria* sp A19 may be beneficial to host plants in providing defense against the adverse effects of abiotic stressors [107,108]. *A*. *fumigatus* associated with soybean plants mitigated adverse effects of saline stress; since the endophyte produced gibberellin and regulated the effects of other phytohormones (abscisic acid, jasmonic and salicylic acid), demonstrating efficiency to promote the host growth [108].

In addition to synthesizing IAA, A19 and P42 strains produced siderophores (S3 Table). Siderophores are heavy iron and metal chelating agents, which may decrease metal phytotoxicity and increase bioavailability [35]. The inoculation of *Brassica napus* with *Fusarium* sp. CBRF44, producer of siderophores and IAA, provided an increase in the dry biomass of the host, besides promoting the phytoextraction of Pb^+2^ and Cd^+2^ [19].

*Massariosphaeria* sp. was the taxon environment indicator with mercury and was the most isolated endophytic species of A. *fluminensis* in this condition. In addition, the species together with *Colletotrichum* sp. and Lindgomycetaceae 1 were the most abundant endophytes, disregarding the type of host and environment (S2 Table). The interaction between host and root endophytes has been shown important for the maintenance of these partners in stressful environments, such as those contaminated with heavy metals, demonstrating the importance of this mutual association [109].

In addition to siderophore and IAA production, the isolates demonstrated the ability to produce hydrolytic enzymes (Table 3; S3 Table). These functional traits are capable of altering the toxicity or bioavailability of the contaminants, contributing to plant growth [110–113]. Bioavailability is influenced by the absorption of metals by the mycelium of arbuscular mycorrhizal fungi, decreasing absorption by plant cells [114].

It should be mentioned that *Massariosphaeria* sp A19, Lindgomycetaceae 1 P87 and *C*. *uredinicola* A72 were stimulated by Hg^+2^ (TI > 1), demonstrating that metal tolerant strains protect their hosts against heavy metal toxicity [115,116]. Endophytic fungi alter physiological functions of the host plant allowing it to resist stresses caused by heavy metals or other toxic agents, favoring the adaptation of the host to the environment and facilitating host establishment [19,20,29].

In plants, mercury ions can replace the magnesium of photosynthetic pigments and interfere with electron transport in chloroplasts, affecting photosynthesis and oxidative metabolism [117,118], which may explain reduction of the growth of *A*. *fluminensis* under the effect of mercury, especially in the reduction of the chlorophyll (approximately 70%) in non-inoculated plants.

The endophytic fungi that resulted in higher GPE indices resulted in higher chlorophyll content in the host, especially *Massariosphaeria* sp. A19, demonstrating that these microorganisms are beneficial under adverse conditions. This same correlation can be observed in *Solanum nigrum* inoculated with endophytic fungi growing in substrate contaminated by cadmium [20].

Tolerant endophytic fungi have been shown to be important tools for optimizing phytoremediation in metal contaminated areas. These microorganisms act by different mechanisms to moderate the toxic effects of the contaminan, resulting in plant growth promotion [54,64].

Some strains, although Hg^+2^ tolerant, significantly reduced the growth of *A*. *fluminensis* (Table 3). Of these, *F*. *oxisporum* and *C*. *rhizophaga*, have been described as plant pathogens [119,120].

## Conclusion

The data obtained in this research confirm the hypothesis that soil contamination by mercury alters the community structure of root endophytic fungi, whether in composition, abundance or species richness. The presence or absence of mercury in the soil alters the functional traits profile of the endophytic fungal community. Tolerance to multiple heavy metals was not associated with tolerance to mercury, rather tolerant strains of endophytes to Cd^+2^, Pb^+2^ and Zn^+2^ were found in both environments. *A*. *fluminensis* depends on its endophytic fungi for resistance to mercury,. Inoculation of *A*. *fluminensis* with sress tolerance endophytic communities may be an important strategy for *in situ* phytoremediation. Future experiments will be necessary to understand the resistance mechanisms of endophytic fungi to the stresses caused by heavy metals and to evaluate the effectiveness of endophyte-assisted phytoremediation.

## Supporting information

S1 Fig Classes of endophytic fungi isolated. Strains obtained from *Polygonum acuminatum* and *Aeschynomene fluminensis* from contaminated (+PHg and +AHg) and uncontaminated areas (-PHg and -AHg).
(TIF)

S2 Fig Composition of endophytic fungal community. Strains obtained from *Polygonum acuminatum* (P) and *Aeschynomene fluminensis* (A) from contaminated (+Hg) and uncontaminated (-Hg) areas (Order: A: Family: B; Genera:C).
(TIF)

S3 Fig Dissimilarity dendogram (Bray-Curtis) for the four isolated endophytic fungi community. Strains obtained from *Polygonum acuminatum* and *Aeschynomene fluminensis* from contaminated (+PHg and +AHg) and uncontaminated areas (-PHg and -AHg).
(TIF)

S1 Table Macro, micronutrients and mercury at collection places. (DOCX)

S2 Table Endophytic fungal community and tolerance to mercury. Strains obtained from *Polygonum acuminatum* and *Aeschynomene fluminensis* from contaminated (+PHg and +AHg) and uncontaminated areas (-PHg and -AHg).
(DOCX)

S3 Table Qualitative and quantitative analysis of functional traits of the endophytic fungi community. Strains obtained from *Polygonum acuminatum* and *Aeschynomene fluminensis* from contaminated (+PHg and +AHg) and uncontaminated areas (-PHg and -AHg).
(DOCX)
