# Supplementary figures and images for: Endophytic fungal communities of Polygonum acuminatum and Aeschynomene fluminensis are influenced by soil mercury contamination
Source: PLoS One. 2017 Jul 25;12(7):e0182017. doi: 10.1371/journal.pone.0182017 (PMC5526616; doi:10.1371/journal.pone.0182017)

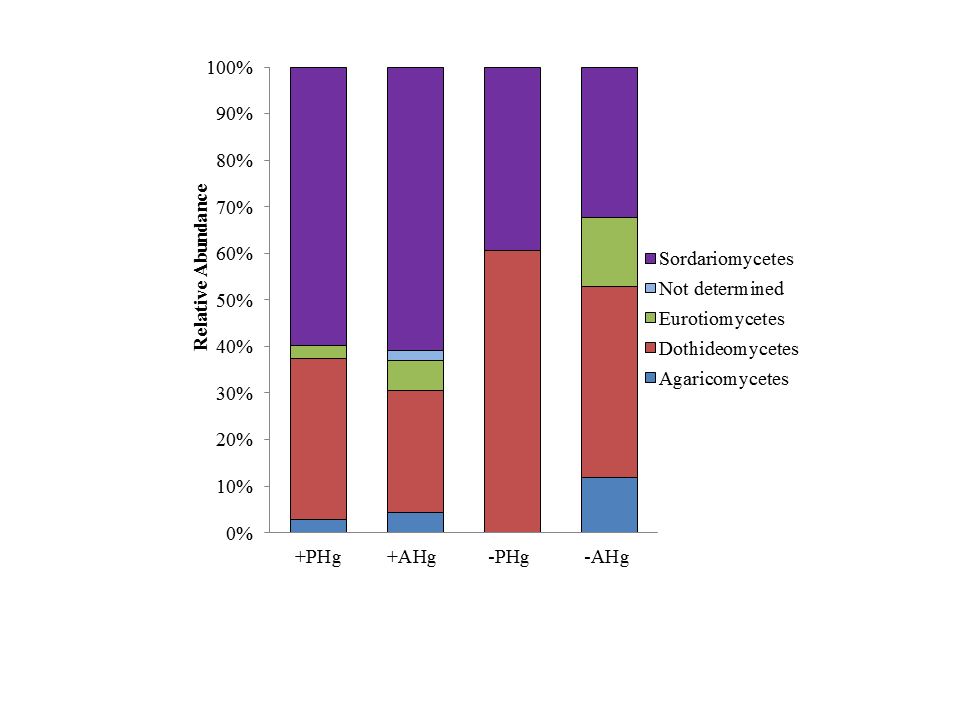

Supplement: S1 Fig — Strains obtained from Polygonum acuminatum and Aeschynomene fluminensis from contaminated (+PHg and +AHg) and uncontaminated areas (-PHg and -AHg). (TIF) [file pone.0182017.s001.tif]

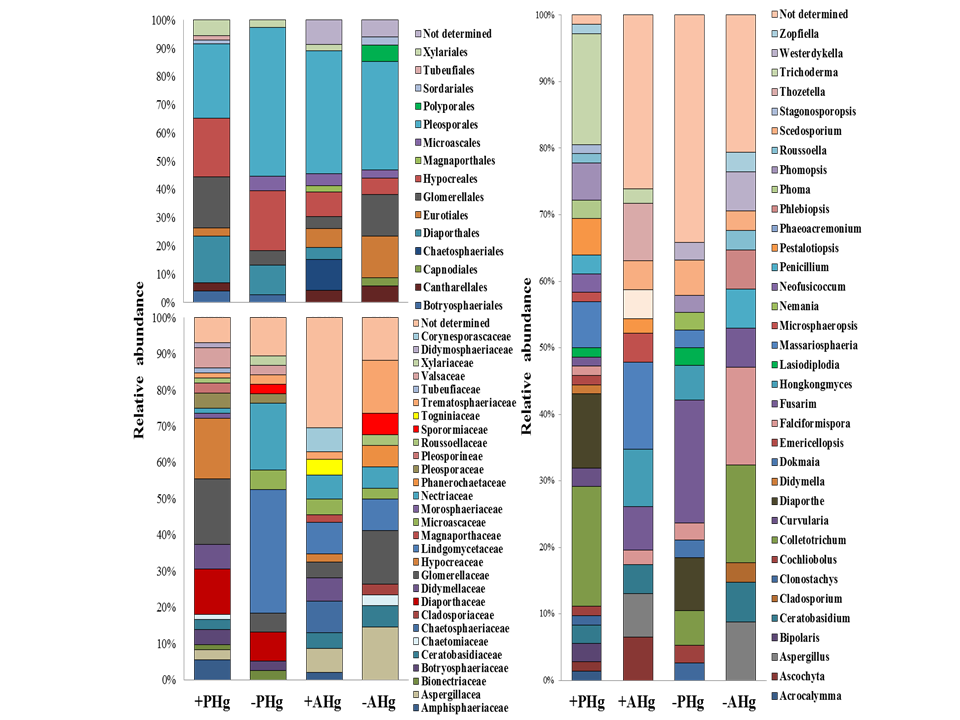

Supplement: S2 Fig — Strains obtained from Polygonum acuminatum (P) and Aeschynomene fluminensis (A) from contaminated (+Hg) and uncontaminated (-Hg) areas (Order: A: Family: B; Genera:C). (TIF) [file pone.0182017.s002.tif]

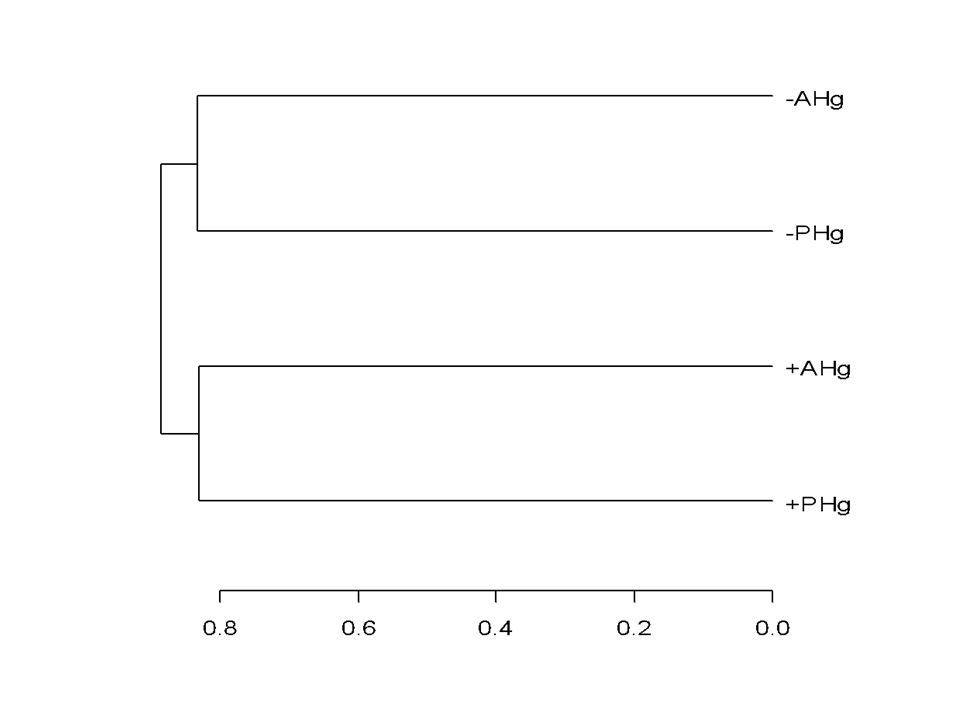

Supplement: S3 Fig — Strains obtained from Polygonum acuminatum and Aeschynomene fluminensis from contaminated (+PHg and +AHg) and uncontaminated areas (-PHg and -AHg). (TIF) [file pone.0182017.s003.tif]
