# Supplementary material for: Endophytic fungal communities of Polygonum acuminatum and Aeschynomene fluminensis are influenced by soil mercury contamination
Source: PLoS One. 2017 Jul 25;12(7):e0182017. doi: 10.1371/journal.pone.0182017 (PMC5526616; doi:10.1371/journal.pone.0182017)
Supplement: S1 Table — (DOCX) [file pone.0182017.s004.docx]

| **Variables** | **Units** | **+Hg Site** | **--Hg Site 1** | **-Hg Site 2** |
| --- | --- | --- | --- | --- |
|  |  | S"16°15'42.7" W"056°38'43.6" | S"16°21'19.7" W"056°20'13.9" | S"16°15'51.3" W"056°38'54.3" |
| **pH** |  | 5,2 | 6,3 | 5,1 |
| **Hg** | mmg.kg^-1^ | 3,24 | 0,0017 | 0,0017 |
| **P** | mg.dm^-3^ | 5,5 | 4,5 | 2 |
| **K** |  | 41 | 40,3 | 37 |
| **Zn** |  | 7,2 | 4,7 | 0,7 |
| **Cu** |  | 3,1 | 2,3 | 0,5 |
| **Fe** |  | 321 | 310 | 116 |
| **Mn** |  | 145,2 | 136,5 | 28,8 |
| **B** |  | 0,4 | 0,4 | 0,2 |
| **S** |  | 11,6 | 11,5 | 6,1 |
| **Ca** | cmol_c_.dm^-3^ | 1 | 3,1 | 0,6 |
| **Mg** |  | 0,4 | 1,6 | 0,3 |
| **Al** |  | 0,4 | 0 | 0,6 |
| **H** |  | 3,4 | 3,1 | 3,9 |
| **Sand** | g.kg^-1^ | 623 | 656 | 690 |
| **Silte** |  | 110 | 67 | 66 |
| **Clay** |  | 267 | 277 | 244 |
