# Supplementary material for: Endophytic fungal communities of Polygonum acuminatum and Aeschynomene fluminensis are influenced by soil mercury contamination
Source: PLoS One. 2017 Jul 25;12(7):e0182017. doi: 10.1371/journal.pone.0182017 (PMC5526616; doi:10.1371/journal.pone.0182017)
Supplement: S2 Table — Strains obtained from Polygonum acuminatum and Aeschynomene fluminensis from contaminated (+PHg and +AHg) and uncontaminated areas (-PHg and -AHg). (DOCX) [file pone.0182017.s005.docx]

| **Species (Blast %)** | **Total** | **+PHg** | **+AHg** | **-PHg** | **-AHg** | **Mercury tolerance** | |
| --- | --- | --- | --- | --- | --- | --- | --- |
|  | **Relative abundance (%)** | | | | | **Strain** | **TI** |
| ^1^Ascomycota (82) | 0.53 | 0.00 | 2.17 | 0.00 | 0.00 | A17* | 1.46 |
| *Acrocalymma vagum* (100) | 0.53 | 1.39 | 0.00 | 0.00 | 0.00 | P18* | 0.90 |
| *Ascochyta medicaginicola* (99) | 2.11 | 1.39 | 6.52 | 0.00 | 0.00 | A9* | 0.93 |
| *Aspergillus japonicus* (99) | 0.53 | 0.00 | 2.17 | 0.00 | 0.00 | A32* | 1.00 |
| *Aspergillus* sp1 (99) | 0.53 | 0.00 | 2.17 | 0.00 | 0.00 | A51* | 0.94 |
| *Aspergillus* sp2 (99) | 2.11 | 0.00 | 2.17 | 0.00 | 8.82 | A31* | 0.93 |
| *Bipolaris setariae* (99) | 0.53 | 1.39 | 0.00 | 0.00 | 0.00 | P4 | 0.89 |
| *Bipolaris sorokiniana* (99) | 0.53 | 1.39 | 0.00 | 0.00 | 0.00 | P61 | 0.71 |
| *Ceratobasidium* sp.1 (95) | 1.58 | 0.00 | 2.17 | 0.00 | 5.88 | A50 | 0.78 |
| *Ceratobasidium* sp.2 (99) | 1.58 | 2.78 | 2.17 | 0.00 | 0.00 | P112 | 0.78 |
| Chaetosphaeriales (99) | 0.53 | 0.00 | 2.17 | 0.00 | 0.00 | A24 | 0.00 |
| *Cladosporium uredinicola* (100) | 0.53 | 0.00 | 0.00 | 0.00 | 2.94 | A72* | 1.24 |
| *Clonostachys rhizophaga* (99) | 0.53 | 0.00 | 0.00 | 2.63 | 0.00 | P89* | 0.98 |
| *Clonostachys rogersoniana* (99) | 0.53 | 1.39 | 0.00 | 0.00 | 0.00 | P62* | 1.03 |
| *Cochliobolus geniculatus* (99) | 0.53 | 1.39 | 0.00 | 0.00 | 0.00 | P59* | 0.95 |
| *Cochliobolus* sp. (99) | 0.53 | 0.00 | 0.00 | 2.63 | 0.00 | P86* | 2.06 |
| *Colletotrichum gloeosporioides* (100) | 3.16 | 5.56 | 0.00 | 5.26 | 0.00 | P24* | 0.97 |
| *Colletotrichum* sp. (100) | 7.37 | 12.50 | 0.00 | 0.00 | 14.71 | P42* | 1.03 |
| *Curvularia geniculata* (100) | 1.05 | 2.78 | 0.00 | 0.00 | 0.00 | P1* | 0.95 |
| *Diaporthe miriciae* (99) | 0.53 | 0.00 | 0.00 | 2.63 | 0.00 | P96* | 0.94 |
| *Diaporthe phaseolorum* (99) | 5.26 | 11.11 | 0.00 | 5.26 | 0.00 | P43 | 0.76 |
| *Didymella subherbarum* (99) | 0.53 | 1.39 | 0.00 | 0.00 | 0.00 | P51 | 0.45 |
| *Dokmaia* sp. (100) | 0.53 | 0.00 | 0.00 | 2.63 | 0.00 | P113* | 0.93 |
| *Emericellopsis* sp. (99) | 0.53 | 1.39 | 0.00 | 0.00 | 0.00 | P54 | 0.83 |
| *Falciformispora* sp.1 (99) | 2.11 | 0.00 | 0.00 | 2.63 | 8.82 | A49* | 0.98 |
| *Falciformispora* sp.2 (99) | 1.05 | 1.39 | 2.17 | 0.00 | 0.00 | A30 | 0.50 |
| *Falciformispora* sp.3 (98) | 1.05 | 0.00 | 0.00 | 0.00 | 5.88 | A76 | 0.35 |
| *Fusarium decemcellulare* (99) | 0.53 | 0.00 | 2.17 | 0.00 | 0.00 | A3 | 0.66 |
| *Fusarium oxysporum* (99) | 4.21 | 0.00 | 0.00 | 15.79 | 5.88 | A64* | 0.96 |
| *Fusarium proliferatum* (99) | 0.53 | 1.39 | 0.00 | 0.00 | 0.00 | P63 | 0.85 |
| *Fusarium solani* (100) | 1.58 | 0.00 | 4.35 | 2.63 | 0.00 | P83 | 0.73 |
| Glomerellaceae (91) | 1.05 | 0.00 | 4.35 | 0.00 | 0.00 | A43 | 0.47 |
| *Hongkongmyces* sp.1 (96) | 1.58 | 0.00 | 6.52 | 0.00 | 0.00 | A27 | 0.84 |
| *Hongkongmyces* sp.2 (96) | 0.53 | 0.00 | 2.17 | 0.00 | 0.00 | A40 | 0.83 |
| *Hongkongmyces pedis* (99) | 1.05 | 0.00 | 0.00 | 5.26 | 0.00 | P107* | 0.93 |
| *Lasiodiplodia pseudotheobromae* (100) | 1.05 | 1.39 | 0.00 | 2.63 | 0.00 | P88 | 0.71 |
| Lindgomycetaceae 1 (94) | 6.84 | 0.00 | 0.00 | 28.95 | 5.88 | P87* | 1.33 |
| Lindgomycetaceae 2 (94) | 0.53 | 0.00 | 0.00 | 0.00 | 2.94 | A73 | 0.72 |
| Magnaporthaceae (99) | 0.53 | 0.00 | 2.17 | 0.00 | 0.00 | A23 | 0.45 |
| *Massariosphaeria* sp. (99) | 6.32 | 6.94 | 13.04 | 2.63 | 0.00 | A19* | 1.52 |
| *Microsphaeropsis arundinis* (99) | 1.58 | 1.39 | 4.35 | 0.00 | 0.00 | A36* | 1.02 |
| *Nemania* sp. (99) | 0.53 | 0.00 | 0.00 | 2.63 | 0.00 | P72 | 0.44 |
| *Neofusicoccum parvum* (98) | 1.05 | 2.78 | 0.00 | 0.00 | 0.00 | P25 | 0.64 |
| *Penicillium janthinellum (*99) | 1.05 | 0.00 | 0.00 | 0.00 | 5.88 | A56* | 1.04 |
| *Penicillium oxalicum* (99) | 0.53 | 1.39 | 0.00 | 0.00 | 0.00 | P32* | 1.07 |
| *Penicillium* sp. (99) | 0.53 | 1.39 | 0.00 | 0.00 | 0.00 | P33 | 0.83 |
| *Pestalotiopsis* sp. (99) | 2.63 | 5.56 | 2.17 | 0.00 | 0.00 | P10 | 0.33 |
| *Phaeoacremonium* sp.1 (99) | 0.53 | 0.00 | 2.17 | 0.00 | 0.00 | A14 | 0.47 |
| *Phaeoacremonium* sp.2 (99) | 0.53 | 0.00 | 2.17 | 0.00 | 0.00 | A26 | 0.59 |
| *Phlebiopsis* sp. (99) | 1.05 | 0.00 | 0.00 | 0.00 | 5.88 | A75* | 1.00 |
| *Phoma* sp.1 (99) | 0.53 | 1.39 | 0.00 | 0.00 | 0.00 | P7 | 0.55 |
| *Phoma* sp.2 (99) | 0.53 | 1.39 | 0.00 | 0.00 | 0.00 | P67* | 0.95 |
| *Phomopsis* sp. (99) | 2.63 | 5.56 | 0.00 | 2.63 | 0.00 | P49 | 0.30 |
| Pleosporales 1 (95) | 2.11 | 0.00 | 2.17 | 5.26 | 2.94 | P74 | 0.85 |
| Pleosporales 2 (99) | 0.53 | 0.00 | 0.00 | 0.00 | 2.94 | A59 | 0.79 |
| Pleosporales 3 (100) | 1.58 | 0.00 | 6.52 | 0.00 | 0.00 | A11 | 0.24 |
| *Roussoella* sp (99) | 1.05 | 1.39 | 0.00 | 0.00 | 2.94 | A68 | 0.71 |
| *Scedosporium apiospermum* (100) | 1.05 | 0.00 | 2.17 | 0.00 | 2.94 | A42* | 0.92 |
| *Scedosporium boydii* (99) | 1.58 | 0.00 | 2.17 | 5.26 | 0.00 | A38* | 0.91 |
| ^1^Sordariomycetes 1 (85) | 1.58 | 0.00 | 6.52 | 0.00 | 0.00 | A18* | 0.94 |
| ^1^Sordariomycetes 2 (86) | 1.05 | 0.00 | 0.00 | 0.00 | 5.88 | A65 | 0.82 |
| *Stagonosporopsis cucurbitacearum* (97) | 0.53 | 1.39 | 0.00 | 0.00 | 0.00 | P26 | 0.85 |
| *Thozetella* sp. (97) | 2.11 | 0.00 | 8.70 | 0.00 | 0.00 | A10 | 0.42 |
| *Trichoderma brevicompactum* (100) | 5.79 | 13.89 | 2.17 | 0.00 | 0.00 | P35* | 0.97 |
| *Trichoderma harzianum* (99) | 1.05 | 2.78 | 0.00 | 0.00 | 0.00 | P39 | 0.58 |
| Tubeufiaceae (94) | 0.53 | 1.39 | 0.00 | 0.00 | 0.00 | P52 | 0.17 |
| *Westerdykella* sp.1 (99) | 0.53 | 0.00 | 0.00 | 2.63 | 0.00 | P71* | 0.95 |
| *Westerdykella* sp.2 (99) | 1.05 | 0.00 | 0.00 | 0.00 | 5.88 | A47* | 1.05 |
| *Zopfiella latipes (99)* | 1.05 | 1.39 | 0.00 | 0.00 | 2.94 | P55 | 0.49 |
| **Total number of isolates** | **190** | **72** | **46** | **38** | **34** | **-** | **-** |

1 Sequence identified by the partial region of the β-Tubulin gene

* Selected strains for inoculation in A. fluminensis
