# Supplementary material for: Endophytic fungal communities of Polygonum acuminatum and Aeschynomene fluminensis are influenced by soil mercury contamination
Source: PLoS One. 2017 Jul 25;12(7):e0182017. doi: 10.1371/journal.pone.0182017 (PMC5526616; doi:10.1371/journal.pone.0182017)
Supplement: S3 Table — Strains obtained from Polygonum acuminatum and Aeschynomene fluminensis from contaminated (+PHg and +AHg) and uncontaminated areas (-PHg and -AHg). (DOCX) [file pone.0182017.s006.docx]

|  | **Species/Strain** | **Enzymes** | | | | | **Antibiosis** | | **Index of tolerance** | | | **Sid** | **IAA** |
| --- | --- | --- | --- | --- | --- | --- | --- | --- | --- | --- | --- | --- | --- |
|  |  | Prot | Ami | Cel | Lig | Lip | G+ | G- | Pb^+2^ | Zn^+2^ | Cd^+2^ |  |  |
| +AHg | *A. japonicus* A32 | ⁺ | ⁺ | ⁺⁺⁺ | ⁺ | ⁺ | ⁻ | ⁻ | 0.68 | 0.00 | 0.00 | ⁺⁺ | 0.00 |
| +AHg | *A. medicaginicola* A9 | ⁻ | ⁺ | ⁻ | ⁺⁺ | ⁻ | ⁻ | ⁻ | 0.89 | 0.46 | 0.49 | ⁻ | 0.00 |
| +PHg | *A. medicaginicola* P60 | ⁻ | ⁻ | ⁻ | ⁺ | ⁻ | ⁻ | ⁻ | 0.99 | 0.45 | 0.30 | ⁺⁺ | 4.23 |
| +PHg | *A. vagum* P18 | ⁻ | ⁺ | ⁻ | ⁺⁺ | ⁻ | ⁺⁺ | ⁻ | 0.86 | 0.39 | 0.30 | ⁻ | 0.90 |
| +AHg | Ascomycota A17 | ⁻ | ⁻ | ⁺ | ⁺ | ⁻ | ⁻ | ⁻ | 1.03 | 0.72 | 0.00 | ⁻ | 5.40 |
| +AHg | *Aspergillus* sp.1 A31 | ⁺ | ⁺ | ⁺ | ⁺ | ⁻ | ⁻ | ⁻ | 0.50 | 0.57 | 0.00 | ⁻ | 8.27 |
| +AHg | *Aspergillus* sp.2 A25 | ⁺ | ⁺ | ⁺ | ⁺ | ⁻ | ⁺⁺ | ⁺ | 2.69 | 0.00 | 0.00 | ⁻ | 26.1 |
| -AHg | *Aspergillus* sp.2 A51 | ⁻ | ⁺⁺ | ⁺⁺ | ⁺ | ⁺⁺⁺ | ⁻ | ⁻ | 0.68 | 0.63 | 0.00 | ⁻ | 3.73 |
| +PHg | *B. setariae* P4 | ⁻ | ⁺⁺⁺ | ⁻ | ⁻ | ⁻ | ⁻ | ⁻ | 0.37 | 1.06 | 0.00 | ⁻ | 0.00 |
| +PHg | *B. sorokiniana* P61 | ⁻ | ⁺⁺⁺ | ⁻ | ⁻ | ⁻ | ⁺⁺ | ⁻ | 0.92 | 0.33 | 0.00 | ⁻ | 25.20 |
| +PHg | *C. geniculata* P1 | ⁺⁺ | ⁺⁺ | ⁻ | ⁻ | ⁻ | ⁻ | ⁻ | 0.72 | 0.42 | 0.20 | ⁻ | 35.10 |
| +PHg | *C. geniculatus* P59 | ⁺ | ⁺⁺ | ⁻ | ⁺⁺ | ⁻ | ⁺⁺ | ⁻ | 0.56 | 0.27 | 0.31 | ⁻ | 0.00 |
| -PHg | *C. gloeosporioides* P106 | ⁺⁺⁺ | ⁺ | ⁻ | ⁺⁺⁺ | ⁺⁺⁺ | ⁻ | ⁻ | 0.81 | 0.43 | 0.19 | ⁻ | 6.29 |
| +PHg | *C. gloeosporioides* P24 | ⁺ | ⁺⁺ | ⁻ | ⁺⁺⁺ | ⁺ | ⁻ | ⁻ | 0.00 | 0.00 | 0.18 | ⁺ | 14.4 |
| -PHg | *C. rhizophaga* P89 | ⁻ | ⁺ | ⁺ | ⁺⁺ | ⁻ | ⁺⁺ | ⁺ | 1.05 | 0.65 | 0.33 | ⁻ | 0.00 |
| +PHg | *C. rogersoniana* P62 | ⁺ | ⁺ | ⁺ | ⁻ | ⁻ | ⁻ | ⁻ | 0.85 | 0.51 | 0.54 | ⁻ | 13.2 |
| -AHg | *C. uredinicola* A72 | ⁺⁺⁺ | ⁺ | ⁺ | ⁺ | ⁻ | ⁻ | ⁻ | 0.70 | 0.00 | 0.00 | ⁻ | 0.00 |
| +AHg | *Ceratobasidium* sp.1 A34 | ⁻ | ⁺⁺ | ⁻ | ⁻ | ⁻ | ⁻ | ⁻ | 1.00 | 0.00 | 0.00 | ⁻ | 2.31 |
| -AHg | *Ceratobasidium* sp.1 A50 | ⁺ | ⁺ | ⁻ | ⁻ | ⁺⁺⁺ | ⁻ | ⁻ | 1.00 | 0.85 | 0.00 | ⁺⁺⁺ | 17.20 |
| +AHg | *Ceratobasidium* sp.2 A1 | ⁻ | ⁻ | ⁺⁺⁺ | ⁻ | ⁺⁺⁺ | ⁻ | ⁻ | 0.88 | 0.26 | 0.34 | ⁺ | 0.00 |
| +PHg | *Ceratobasidium* sp.2 P112 | ⁻ | ⁺⁺⁺ | ⁺⁺ | ⁻ | ⁻ | ⁻ | ⁻ | 0.57 | 0.00 | 0.00 | ⁺⁺⁺ | 3.31 |
| +AHg | Chaetosphaeriales A24 | ⁻ | ⁺ | ⁻ | ⁺⁺⁺ | ⁻ | ⁻ | ⁺ | 0.89 | 0.65 | 0.00 | ⁻ | 0.00 |
| -PHg | *Cochliobolus sp.* P86 | ⁻ | ⁺⁺ | ⁻ | ⁻ | ⁻ | ⁻ | ⁻ | 0.81 | 0.47 | 0.44 | ⁻ | 0.00 |
| -AHg | *Colletotrichum* sp. A46 | ⁻ | ⁺ | ⁻ | ⁺⁺⁺ | ⁻ | ⁻ | ⁻ | 0.89 | 0.55 | 0.51 | ⁻ | 14.40 |
| +PHg | *Colletotrichum* sp. P42 | ⁺ | ⁺ | ⁺ | ⁻ | ⁻ | ⁻ | ⁻ | 0.78 | 0.46 | 0.00 | ⁺ | 0.00 |
| +AHg | Corynesporascaceae A11 | ⁻ | ⁺ | ⁻ | ⁺ | ⁻ | ⁻ | ⁻ | 0.66 | 0.47 | 0.25 | ⁻ | 15.20 |
| -PHg | *D. miriciae* P96 | ⁺ | ⁺⁺ | ⁻ | ⁺ | ⁻ | ⁻ | ⁻ | 1.00 | 0.00 | 0.00 | ⁻ | 25.20 |
| +PHg | *D. phaseolorum*  P48 | ⁺ | ⁺⁺ | ⁺⁺ | ⁺⁺ | ⁻ | ⁻ | ⁻ | 1.00 | 0.00 | 0.11 | ⁻ | 58.5 |
| -PHg | *D. phaseolorum* P79 | ⁻ | ⁺⁺⁺ | ⁺⁺⁺ | ⁺⁺⁺ | ⁻ | ⁻ | ⁻ | 0.99 | 0.00 | 0.35 | ⁻ | 4.50 |
| +PHg | *D. subherbarum* P51 | ⁻ | ⁺ | ⁻ | ⁺ | ⁺ | ⁻ | ⁻ | 0.82 | 0.00 | 0.00 | ⁻ | 0.00 |
| -PHg | *Dokmaia* sp. P113 | ⁺ | ⁻ | ⁻ | ⁻ | ⁻ | ⁻ | ⁻ | 0.76 | 0.35 | 0.00 | ⁻ | 1.54 |
| +PHg | *Emericellopsis* sp. P54 | ⁺⁺⁺ | ⁺⁺ | ⁺ | ⁺⁺⁺ | ⁺⁺ | ⁺⁺ | ⁻ | 0.73 | 0.00 | 0.43 | ⁻ | 8.57 |
| +AHg | *F. decemcellulare* A3 | ⁻ | ⁺ | ⁺ | ⁺⁺ | ⁻ | ⁻ | ⁻ | 0.83 | 0.19 | 0.19 | ⁺ | 2.70 |
| -AHg | *F. oxysporum* A64 | ⁻ | ⁺ | ⁺ | ⁻ | ⁺⁺⁺ | ⁻ | ⁻ | 0.88 | 0.42 | 0.11 | ⁻ | 6.30 |
| -PHg | *F. oxysporum* P80 | ⁺ | ⁺⁺⁺ | ⁻ | ⁻ | ⁺⁺ | ⁻ | ⁻ | 0.94 | 0.15 | 0.00 | ⁻ | 47.70 |
| +PHg | *F. proliferatum* P63 | ⁺⁺ | ⁺⁺⁺ | ⁻ | ⁺ | ⁺⁺⁺ | ⁻ | ⁻ | 0.84 | 0.00 | 0.00 | ⁻ | 31.80 |
| +AHg | *F. solani* A37 | ⁻ | ⁺⁺ | ⁺⁺ | ⁺ | ⁺⁺⁺ | ⁻ | ⁻ | 0.91 | 0.28 | 0.00 | ⁺⁺ | 9.00 |
| -PHg | *F. solani* P83 | ⁻ | ⁺⁺ | ⁺⁺ | ⁻ | ⁺⁺⁺ | ⁻ | ⁻ | 0.83 | 0.39 | 0.00 | ⁺⁺⁺ | 10.80 |
| -AHg | *Falciformispora* sp.1 A49 | ⁻ | ⁺ | ⁺⁺⁺ | ⁺ | ⁻ | ⁻ | ⁻ | 0.67 | 0.00 | 0.00 | ⁺⁺ | 0.00 |
| -PHg | *Falciformispora* sp.1 P92 | ⁻ | ⁺ | ⁻ | ⁺⁺ | ⁻ | ⁻ | ⁻ | 1.03 | 0.72 | 0.36 | ⁻ | 0.00 |
| +AHg | *Falciformispora* sp.2 A30 | ⁺⁺ | ⁻ | ⁻ | ⁻ | ⁻ | ⁻ | ⁻ | 1.07 | 0.79 | 0.78 | ⁺ | 0.00 |
| -AHg | *Falciformispora* sp.3 A76 | ⁺ | ⁻ | ⁻ | ⁺⁺ | ⁻ | ⁻ | ⁻ | 0.90 | 0.90 | 1.20 | ⁻ | 0.00 |
| +PHg | *Falciformispora* sp.3 P56 | ⁻ | ⁺ | ⁺ | ⁻ | ⁻ | ⁻ | ⁻ | 0.68 | 0.00 | 0.00 | ⁻ | 0.00 |
| +AHg | Glomerellaceae A43 | ⁻ | ⁻ | ⁻ | ⁻ | ⁻ | ⁺ | ⁺ | 0.44 | 0.00 | 0.00 | ⁻ | 0.33 |
| -PHg | *H. pedis* P107 | ⁻ | ⁺ | ⁻ | ⁻ | ⁻ | ⁻ | ⁻ | 0.67 | 0.21 | 0.58 | ⁺ | 7.20 |
| +AHg | *Hongkongmyces*  sp.1 A40 | ⁻ | ⁺ | ⁻ | ⁺ | ⁻ | ⁻ | ⁻ | 1.02 | 0.41 | 0.76 | ⁺ | 12.60 |
| +AHg | *Hongkongmyces*  sp.2 A27 | ⁻ | ⁻ | ⁻ | ⁺⁺⁺ | ⁻ | ⁻ | ⁻ | 1.06 | 0.48 | 0.76 | ⁻ | 0.82 |
| +PHg | *L. pseudotheobromae* P58 | ⁻ | ⁺ | ⁻ | ⁻ | ⁻ | ⁻ | ⁻ | 0.82 | 0.39 | 0.00 | ⁺ | 23.40 |
| -PHg | *L. pseudotheobromae* P88 | ⁻ | ⁻ | ⁻ | ⁻ | ⁻ | ⁻ | ⁻ | 0.55 | 0.40 | 0.00 | ⁺ | 27.90 |
| -AHg | Lindgomycetaceae 1 A54 | ⁻ | ⁺ | ⁻ | ⁺⁺ | ⁻ | ⁻ | ⁻ | 0.54 | 0.54 | 0.53 | ⁺ | 2.70 |
| -PHg | Lindgomycetaceae 1 P87 | ⁻ | ⁺ | ⁺ | ⁻ | ⁻ | ⁺⁺ | ⁻ | 1.05 | 0.62 | 0.31 | ⁻ | 0.00 |
| -AHg | Lindgomycetaceae 2 A73 | ⁻ | ⁻ | ⁻ | ⁻ | ⁻ | ⁻ | ⁻ | 0.80 | 0.47 | 0.55 | ⁺⁺ | 0.00 |
| +AHg | *M. arundinis* A36 | ⁻ | ⁺ | ⁻ | ⁺ | ⁺ | ⁻ | ⁻ | 0.48 | 0.30 | 0.00 | ⁺ | 0.00 |
| +PHg | *M. arundinis* P40 | ⁺ | ⁺⁺⁺ | ⁻ | ⁺⁺ | ⁻ | ⁻ | ⁻ | 0.67 | 0.49 | 0.33 | ⁺⁺ | 75.80 |
| +AHg | Magnaporthaceae A23 | ⁺ | ⁺ | ⁺ | ⁺⁺ | ⁻ | ⁻ | ⁻ | 0.88 | 0.26 | 0.00 | ⁺ | 0.00 |
| +AHg | *Massariosphaeria* sp. A19 | ⁻ | ⁺ | ⁻ | ⁺ | ⁻ | ⁻ | ⁻ | 0.82 | 0.38 | 0.00 | ⁺ | 0.33 |
| +PHg | *Massariosphaeria* sp. P19 | ⁻ | ⁺ | ⁻ | ⁺ | ⁻ | ⁺⁺⁺ | ⁻ | 1.18 | 0.61 | 0.00 | ⁻ | 13.50 |
| -PHg | *Massariosphaeria* sp. P85 | ⁻ | ⁻ | ⁻ | ⁻ | ⁻ | ⁻ | ⁻ | 0.84 | 0.50 | 0.00 | ⁻ | 0.38 |
| +PHg | *N. parvum* P25 | ⁻ | ⁺⁺⁺ | ⁻ | ⁻ | ⁻ | ⁻ | ⁻ | 0.68 | 0.00 | 0.00 | ⁻ | 24.30 |
| -PHg | *Nemania* sp. P72 | ⁺ | ⁺⁺ | ⁻ | ⁺⁺ | ⁺ | ⁻ | ⁻ | 1.00 | 0.00 | 0.15 | ⁻ | 1.80 |
| -AHg | *P. janthinellum* A56 | ⁻ | ⁺⁺ | ⁺⁺ | ⁺ | ⁺⁺⁺ | ⁻ | ⁻ | 0.48 | 0.13 | 0.00 | ⁻ | 0.00 |
| +PHg | *P. oxalicum* P32 | ⁺ | ⁺ | ⁺⁺ | ⁺ | ⁻ | ⁻ | ⁻ | 0.64 | 0.00 | 0.00 | ⁺⁺ | 0.00 |
| +PHg | *Penicillium* sp. P33 | ⁻ | ⁺ | ⁺⁺ | ⁻ | ⁺ | ⁻ | ⁻ | 0.58 | 0.19 | 0.00 | ⁺ | 16.30 |
| +AHg | *Pestalotiopsis* sp. A22 | ⁺ | ⁺ | ⁺ | ⁺⁺ | ⁻ | ⁻ | ⁻ | 1.00 | 0.00 | 0.08 | ⁻ | 10.80 |
| +PHg | *Pestalotiopsis* sp. P10 | ⁻ | ⁺⁺ | ⁻ | ⁺⁺ | ⁺ | ⁻ | ⁻ | 0.76 | 0.00 | 0.00 | ⁺ | 25.20 |
| +AHg | *Phaeoacremonium* sp.1 A14 | ⁻ | ⁺ | ⁻ | ⁺ | ⁻ | ⁻ | ⁺ | 0.68 | 0.48 | 0.00 | ⁺⁺ | 0.00 |
| +AHg | *Phaeoacremonium* sp.2 A26 | ⁻ | ⁺ | ⁻ | ⁺ | ⁻ | ⁻ | ⁻ | 0.85 | 0.37 | 0.00 | ⁺ | 0.00 |
| -AHg | *Phlebiopsis* sp. A75 | ⁺ | ⁺⁺ | ⁺⁺ | ⁻ | ⁻ | ⁻ | ⁻ | 0.94 | 0.00 | 0.00 | ⁻ | 0.00 |
| +PHg | *Phoma* sp 2 P67 | ⁻ | ⁺⁺ | ⁻ | ⁺ | ⁺ | ⁻ | ⁻ | 0.72 | 0.00 | 0.00 | ⁻ | 11.70 |
| +PHg | *Phoma* sp.1 P7 | ⁻ | ⁺ | ⁻ | ⁺⁺ | ⁻ | ⁻ | ⁻ | 0.95 | 0.00 | 0.00 | ⁻ | 0.00 |
| -PHg | *Phomopsis* sp. P102 | ⁻ | ⁺ | ⁺⁺ | ⁺⁺ | ⁻ | ⁻ | ⁻ | 1.00 | 0.00 | 0.00 | ⁻ | 3.60 |
| +PHg | *Phomopsis* sp. P49 | ⁺ | ⁺ | ⁻ | ⁺⁺ | ⁻ | ⁻ | ⁺ | 0.00 | 1.00 | 0.00 | ⁻ | 54.90 |
| -AHg | Pleosporales 1 A80 | ⁻ | ⁻ | ⁻ | ⁻ | ⁻ | ⁻ | ⁻ | 1.00 | 0.62 | 0.55 | ⁻ | 4.23 |
| -PHg | Pleosporales 1 P74 | ⁻ | ⁺ | ⁻ | ⁻ | ⁻ | ⁻ | ⁻ | 0.97 | 0.72 | 0.64 | ⁻ | 3.60 |
| -AHg | Pleosporales 2 A59 | ⁻ | ⁺ | ⁻ | ⁺⁺ | ⁺ | ⁻ | ⁻ | 1.09 | 0.98 | 0.00 | ⁺ | 9.00 |
| -AHg | *Roussoella* sp. A68 | ⁻ | ⁺⁺ | ⁻ | ⁻ | ⁻ | ⁺⁺⁺ | ⁻ | 0.64 | 0.42 | 0.55 | ⁺ | 0.00 |
| +PHg | *Roussoella* sp. P70 | ⁻ | ⁺ | ⁻ | ⁻ | ⁻ | ⁻ | ⁻ | 0.81 | 0.40 | 0.92 | ⁺ | 0.90 |
| +AHg | *S. apiospermum* A42 | ⁻ | ⁺ | ⁻ | ⁻ | ⁺ | ⁻ | ⁻ | 0.87 | 0.47 | 0.00 | ⁻ | 2.31 |
| -AHg | *S. apiospermum* A62 | ⁺ | ⁻ | ⁻ | ⁺⁺⁺ | ⁻ | ⁻ | ⁻ | 0.78 | 0.45 | 0.00 | ⁻ | 5.74 |
| +AHg | *S. boydii* A38 | ⁺ | ⁺ | ⁻ | ⁻ | ⁺ | ⁻ | ⁻ | 0.90 | 0.62 | 0.62 | ⁻ | 0.00 |
| -PHg | *S. boydii* P94 | ⁻ | ⁺ | ⁻ | ⁻ | ⁺ | ⁻ | ⁻ | 0.00 | 0.87 | 0.00 | ⁻ | 0.00 |
| +PHg | *S. cucurbitacearum* P26 | ⁻ | ⁺⁺ | ⁻ | ⁻ | ⁻ | ⁻ | ⁻ | 0.48 | 0.00 | 0.00 | ⁻ | 0.00 |
| +AHg | Sordariomycetes 1 A18 | ⁻ | ⁻ | ⁻ | ⁺⁺ | ⁻ | ⁻ | ⁻ | 0.59 | 0.00 | 0.00 | ⁻ | 0.00 |
| -AHg | Sordariomycetes 2 A65 | ⁻ | ⁻ | ⁻ | ⁺⁺ | ⁻ | ⁻ | ⁻ | 0.96 | 0.39 | 0.00 | ⁻ | 0.00 |
| +AHg | *T. brevicompactum* A35 | ⁻ | ⁺⁺ | ⁻ | ⁻ | ⁻ | ⁻ | ⁻ | 1.00 | 0.69 | 0.16 | ⁺⁺ | 0.00 |
| +PHg | *T. brevicompactum* P35 | ⁻ | ⁺ | ⁻ | ⁻ | ⁺ | ⁻ | ⁻ | 1.00 | 0.76 | 0.26 | ⁺⁺⁺ | 0.00 |
| +PHg | *T. harzianum* P39 | ⁺⁺ | ⁺ | ⁻ | ⁻ | ⁺⁺⁺ | ⁻ | ⁻ | 0.89 | 0.59 | 1.00 | ⁻ | 0.90 |
| +AHg | *Thozetella* sp. A13 | ⁻ | ⁻ | ⁺ | ⁺ | ⁻ | ⁻ | ⁻ | 0.91 | 0.00 | 0.00 | ⁻ | 0.00 |
| +PHg | Tubeufiaceae P52 | ⁻ | ⁺ | ⁻ | ⁻ | ⁻ | ⁻ | ⁻ | 0.84 | 0.31 | 0.32 | ⁻ | 3.60 |
| -PHg | *Westerdykella* sp.1 P71 | ⁻ | ⁺⁺ | ⁺ | ⁻ | ⁻ | ⁺⁺⁺ | ⁺ | 0.83 | 0.00 | 0.00 | ⁺⁺⁺ | 48.60 |
| -AHg | *Westerdykella* sp.2 A47 | ⁻ | ⁺ | ⁻ | ⁺ | ⁻ | ⁻ | ⁻ | 0.96 | 0.31 | 0.00 | ⁻ | 5.40 |
| -AHg | *Z. latipes* A74 | ⁻ | ⁻ | ⁻ | ⁻ | ⁺ | ⁻ | ⁻ | 0.82 | 0.00 | 0.00 | ⁻ | 10.50 |
| +PHg | *Z. latipes* P55 | ⁻ | ⁺ | ⁺ | ⁻ | ⁻ | ⁻ | ⁻ | 0.82 | 0.00 | 0.00 | ⁻ | 2.31 |

Siderophore (SID), indoleacetic acid (IAA), hydrolytic enzymes (Amy- Amylase; Cel- Cellulase, Prot- Protease, Lig- Ligninase), antibiosis against strains of pathogenic G+ bacteria (S. saprophyticus) and G- (E. coli) (Pb^+2^, Zn^+2^and Cd ^+2^) isolated from *Polygonum acuminatum* and *Aeschynomene fluminensis* from contaminated (+PHg and +AHg) and uncontaminated areas (-PHg and -AHg).
